# The impact of Hfq-mediated sRNA-mRNA interactome on the virulence of enteropathogenic *Escherichia coli*

**DOI:** 10.1126/sciadv.abi8228

**Published:** 2021-10-27

**Authors:** Sivan Pearl Mizrahi, Netanel Elbaz, Liron Argaman, Yael Altuvia, Naama Katsowich, Yaakov Socol, Amir Bar, Ilan Rosenshine, Hanah Margalit

**Affiliations:** Department of Microbiology and Molecular Genetics, Institute for Medical Research Israel-Canada, Faculty of Medicine, The Hebrew University of Jerusalem, Jerusalem 9112102, Israel.

## Abstract

Small RNAs (sRNAs) exert their regulation posttranscriptionally by base pairing with their target mRNAs, often in association with the RNA chaperone protein Hfq. Here, integrating RNA-seq–based technologies and bioinformatics, we deciphered the Hfq-mediated sRNA-target interactome of enteropathogenic *Escherichia coli* (EPEC). The emerging network comprises hundreds of sRNA-mRNA pairs, including mRNAs of virulence-associated genes interacting with known sRNAs encoded within the core genome, as well as with newly found sRNAs encoded within pathogenicity islands. Some of the sRNAs affect multiple virulence genes, suggesting they function as hubs of virulence control. We further analyzed one such sRNA hub, MgrR, and one of its targets identified here, the major virulence-associated chaperon, *cesT*. We show that MgrR adjusts the level of EPEC cytotoxicity via regulation of CesT expression. Our results reveal an elaborate sRNA-mRNA interactome controlling the pathogenicity of EPEC and reinforce a role for sRNAs in the control of pathogen-host interaction.

## Teaser

An elaborate network of sRNA-mRNA interactions controls the pathogenicity of enteropathogenic *Escherichia coli.*

## INTRODUCTION

Enteropathogenic *Escherichia coli* (EPEC) is a human bacterial pathogen causing conditions ranging from asymptomatic colonization to persistent life-threatening infantile diarrhea (*1*). The Type III secretion system (T3SS) is the major virulence factor of EPEC, constituting a molecular syringe used by the pathogen to inject dozens of effector proteins into the host cell. These effectors subvert host cell processes to promote host colonization (*2*, *3*). The recruitment of effectors to the T3SS is largely mediated by the CesT multicargo chaperon. An additional important virulence factor of a typical EPEC is the type 4 pilus, termed bundle-forming pilus (BFP), which promotes bacterial aggregation to form microcolonies and efficient attachment of these microcolonies to the host cell (*4*–*6*). The combined functions of BFP and T3SS promote efficient colonization, long-term survival, and rapid growth of the pathogen, despite the host defenses.

EPEC has a core genome that is nearly identical to the genome of the nonpathogenic *E. coli* K-12 strain, but includes ~1000 additional genes, termed here “accessory genome” genes. Most of the accessory genome genes are clustered within chromosomal regions termed “genomic islands,” consisting of prophages and insertion elements. Many of these islands include virulence genes and thus are termed also “pathogenicity islands.” The T3SS and related genes, altogether 41, are organized in 12 transcriptional units clustered within a pathogenicity island termed the locus of enterocyte effacement (LEE) (*7*, *8*). The BFP and its regulators are encoded in two operons, *bfpA-P* and *perABC*, both located in a large plasmid, pMAR2. The transcription of the T3SS and BFP genes is controlled by a complex network including four key regulators: PerA, PerC, Ler, and GrlR-GrlA (*9*). The production of these regulators is subjected to transcriptional and posttranscriptional regulation, where the latter involves the global RNA binding proteins CsrA (*10*, *11*) and Hfq (*12*, *13*), as well as several small RNAs (sRNAs) (*12*).

sRNAs are 50- to 400-nucleotide-long RNA molecules, many of which regulate their gene targets in trans by base pairing with target mRNA (*14*). Often, this base pairing is facilitated by the RNA chaperone protein Hfq, which binds both the sRNA and its target mRNA and promotes their duplex formation (*15*). Recently, it was demonstrated that Hfq and sRNAs modulate the expression of specific T3SS genes of EPEC (*12*, *13*). The sRNAs MgrR and RyhB were reported to directly modulate the expression of GrlR-GrlA (*12*), and Spf (Spot 42) was found to control the translation of genes encoded in the LEE4 operon of the closely related enterohemorrhagic *E. coli* (EHEC) (*11*). However, the global virulence-associated network of Hfq-mediated sRNA-target pairs has not yet been resolved for EPEC. To address this knowledge gap, we applied the RIL-seq (RNA interaction by ligation and sequencing) methodology (*16*) and revealed the EPEC global Hfq-dependent sRNA-mRNA interactome. Our results indicate that some sRNAs, including novel accessory genome–encoded sRNAs, function as hubs that interact with mRNAs of multiple virulence genes. In-depth analysis of a selected sRNA-mRNA interaction demonstrates how the sRNA modulates the cytotoxicity of the pathogen. All the RIL-seq identified interactions can be queried and visualized through a dedicated online database http://RILseqDB.cs.huji.ac.il.

## RESULTS

### Hfq controls the expression of virulence-associated genes

To evaluate the roles of Hfq and sRNAs in virulence, we aimed at comparing the transcriptome of wild-type EPEC with that of an isogenic Δ*hfq* mutant. To this end, we generated a Δ*hfq* mutant and first tested it for production of Tir, the major T3SS effector, and BfpA, the pilin subunit of BFP. For this analysis, we grew the bacteria under two growth conditions: either in Dulbecco’s modified Eagle’s medium at 37°C to mid-exponential growth phase [OD_600_ (optical density at 600 nm), 0.3 to 0.6], or overnight growth of static culture on LB medium at 37°C. The former condition strongly induces the expression of the virulence genes, and the latter represses their expression. These conditions are termed hereinafter “activating” and “nonactivating,” respectively. Our data showed that the levels of Tir and BfpA were elevated in the Δ*hfq* mutant (fig. S1). However, the overexpression of BFP protein due to the lack of Hfq appears to impose stress (*17*), and consequently, rapid elimination of BFP expression either by curing the bacteria of the pMAR2 plasmid or by rendering *perA* (encoding the BFP master regulator) nonfunctional, through a frameshift mutation (fig. S1). These results suggest that Hfq, presumably in concert with sRNAs, acts to prevent overexpression of the T3SS and BFP genes, where overexpression of the latter might lead to instability.

**Fig. 1.**
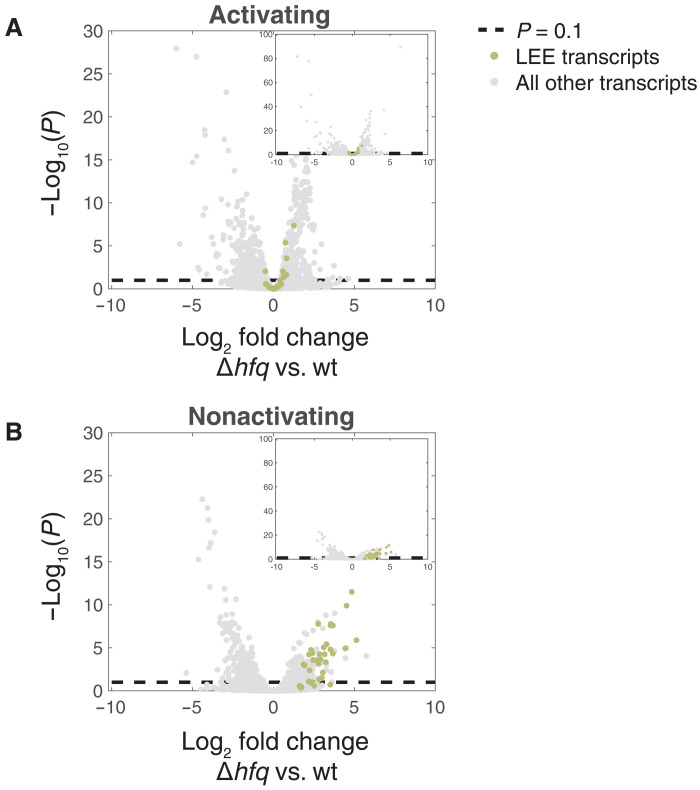
Hfq affects the expression of virulence-associated genes. Volcano plots describing the transcriptome comparisons of EPEC Δ*hfq* versus wild type (wt) under activating (**A**) and nonactivating (**B**) conditions. The change in expression level of each gene is represented as the log_2_ fold change between the expression levels in Δ*hfq* and wt strains, as obtained by applying DESeq2 (*18*) analysis (*x* axis). The statistical significance of the change is represented as −log_10_(*P*) (*y* axis). *P* is the *P* value corrected for multiple hypotheses testing (padj provided by DESeq2). The dashed line marks the statistical significance threshold (*P* = 0.1). LEE-related genes are indicated by green dots, and gray dots represent the rest of the genes. Insets were added to allow viewing genes with very low padj values (highly statistically significant).

To expand this study, we determined the Hfq influence on EPEC’s transcriptome by applying RNA sequencing (RNA-seq) to wild-type and Δ*hfq* mutant, using the mutant version where pMAR2 was stabilized by the acquisition of the *perA* mutation (fig. S1). RNA was extracted from cultures grown under either the activating or nonactivating condition and compared between wild-type and Δ*hfq* strains by DESeq2 (Fig. 1 and tables S1 and S2) (*18*). Note that since we used the double mutant deficient in Hfq and PerA activities, results related to *perA* and *bfp* operons are irrelevant. The transcriptome analyses of the wild-type strain in activating and nonactivating conditions demonstrated that growth under the activating condition promoted the expression of virulence genes (fig. S2). We further noted that Hfq has a clear repressive effect on the expression of the LEE genes (Fig. 1 and table S2) and additional virulence genes encoded in chromosomal locations other than the LEE (table S2). These include, for example, the T3SS effector genes *nleG*, *nleH*, E2348C_2920, located proximal to the *nleG*, and E2348C_0153, encoding a predicted fimbriae protein; E2348C_2914, located in IE5 immediately upstream to the *espC* and encoding a predicted polyketide cyclase; *matA* (*ecpR*), encoding a LuxR-type transcription factor, involved in advancing the transition of *E. coli* from planktonic to adhesive lifestyle (*19*–*21*); and genes associated with the acid resistance response, including *hdeAB*, *gadF*, *gadC*, and *gadX*, some of which are involved also in regulating the LEE genes (table S2) (*22*, *23*). Overall, our results, together with previous reports revalidated here (*12*, *13*, *17*, *24*), support a role for Hfq in regulating multiple virulence genes of EPEC, conceivably together with multiple sRNAs.

### The EPEC Hfq-dependent RNA-RNA interactome

To explore the involvement of sRNAs in EPEC virulence at a global scale, we used RIL-seq, our recently developed methodology for in vivo detection of the Hfq-dependent RNA-RNA interactome (fig. S3) (*16*, *25*). We applied this strategy to analyze EPEC cultures grown under activating and nonactivating conditions. Briefly, bacteria were subjected to ultraviolet cross-linking, lysed, and Hfq along with the bound RNA molecules were immunoprecipitated. The precipitate was then treated with RNA ligase, fusing neighboring RNAs to form chimeric fragments. The resulting RNAs were isolated, sequencing libraries were constructed as described in Melamed *et al.* (*25*), and paired-end sequenced. Our computational pipeline was next used to map the sequenced fragments to EPEC genome (fig. S3), identifying chimeric fragments and determining statistically significant overrepresented chimeric fragments (S-chimeras) as representing putatively interacting RNAs (*16*). In this analysis, we considered reads mapped to several types of genomic elements, most notably coding sequence (CDS); sRNA; tRNA; 5′ and 3′ untranslated regions (5UTR/EST5UTR and 3UTR/EST3UTR, where EST stands for estimated); intergenic region (IGR); intergenic within operon transcript (IGT); and antisense (AS) (see Materials and Methods and Supplementary Methods). For both activating and nonactivating conditions, we applied RIL-seq to three biological replicates, generating a sequencing library for each replicate. Computational analysis for revealing statistically significant RNA pairs was conducted for the library of each replicate separately and for a unified library in which the results of all triplicates per condition were unified. In table S3, we report the interactions that passed the statistical test in the analysis of the unified libraries, and for each interaction we record the number of individual libraries in which it was identified as statistically significant (*26*). Of note, the libraries under both conditions were of similar sizes (table S1). We identified 744 and 971 putative RNA interacting pairs based on the analysis of the unified libraries obtained for the activating and nonactivating conditions, respectively, where 251 interactions were identified under both conditions (table S3, summary tab). The EPEC RIL-seq interaction data can be queried and viewed via our new database RILseqDB (http://RILseqDB.cs.huji.ac.il), a database of Hfq-mediated RNA interactions determined by RIL-seq.

### sRNAs involved in the RNA-RNA interactome of EPEC

#### Many of E. coli K-12 sRNAs and their interactions are conserved in EPEC

Since the core genome of EPEC is nearly identical to the genome of *E. coli* K-12, we first turned to assess whether homologs of known K-12 sRNAs are involved in EPEC RNA-RNA interactome and whether their interactions are conserved (Materials and Methods). We searched by blastn (*27*) the EPEC orthologs of all currently documented sRNAs in K-12 (*28*) and orthologs of recently found sRNAs (*29*) (table S4), and assessed whether they were included in EPEC RIL-seq data. We found that the orthologs of most K-12 sRNAs were included in the RIL-seq–identified RNA-RNA interactome of EPEC (table S4). This regards well-established sRNAs, such as ArcZ and CyaR, as well as recently detected 3′UTR-derived sRNAs, such as MalH (*30*). GlnZ, an sRNA derived from the 3′UTR of *glnA* in *E. coli* K-12 (*28*, *31*), was not detected in our blastn search. However, using several sRNA properties listed hereinafter, we independently found an sRNA candidate derived from the 3′UTR of EPEC *glnA*. As this sRNA candidate has a predicted binding site that is conserved in *E. coli* K-12 and across several bacterial species belonging to Enterobacteriaceae, we believe it is a *glnZ* ortholog (fig. S4). The genomic context of *glnZ* binding site differs among species, i.e., there are large insertions/deletions and the distances of the predicted binding site from the stop codon and the Rho-independent terminator of *glnA* are not fixed (fig. S4). This highlights the conservation variability of 3′UTR-derived sRNAs, where some are evolutionarily conserved throughout their length [e.g., the recently discovered CpxQ (*32*)], while others, such as GlnZ, are only partially conserved.

**Fig. 2.**
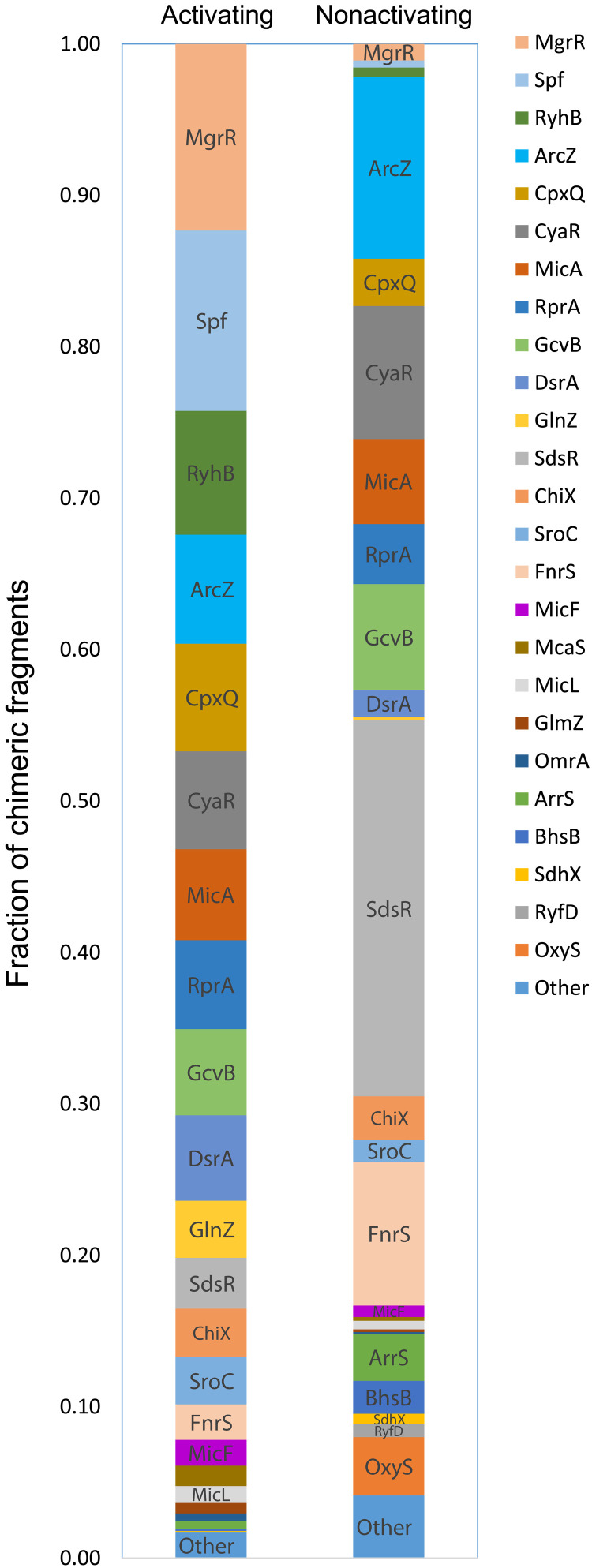
Relative abundance of core-encoded sRNAs in EPEC RIL-seq data. For each sRNA in S-chimeras, the relative abundance of the respective chimeric fragments identified in bacteria grown under the activating and nonactivating conditions is shown. This fraction is obtained by dividing the number of chimeric fragments involving a given sRNA by the total number of chimeric fragments involving core-encoded sRNAs. Numbers were extracted from table S3. Some chimeric fragments involve two sRNAs, in which case the number of chimeric fragments is assigned to each of the two interacting sRNAs and the total is updated accordingly. Thus, the total numbers of chimeric fragments used here are 66,540 and 83,865 for activating and nonactivating conditions, respectively. Twenty-six sRNAs with very low abundances under both conditions (<0.005) are grouped to one category termed as “other.”

It is worth noting that the relative abundance of specific core-encoded sRNAs in the interactome differed between the two conditions (Fig. 2). For example, the sRNAs Spf, RyhB, MgrR, and CpxQ had high relative abundances in the interactome identified under the activating condition, but constituted a relatively low fraction of the interactome identified under the nonactivating condition, suggesting rewiring of the sRNA regulatory network upon infection. In contrast, other sRNAs, such as SdsR and FnrS, had high relative abundances in the interactome identified under the nonactivating condition but not under the activating condition. In general, there is an agreement between the relative fraction of chimeric fragments involving an sRNA and its expression level measured in the RNA-seq experiment (Fig. 2 and fig. S2). Note however that Hfq occupancy of an sRNA can be affected not only by the global sRNA expression levels but also by other factors such as changes in its target abundance and/or in the abundance of other competing sRNAs. The potential link between the abundance of the sRNA under the activating condition and its relation to virulence is exemplified by Spf, with 20-fold higher fraction of chimeric fragments involving Spf in the activating versus the nonactivating condition. Consistently, its expression level is ~5-fold higher in the activating compared to the nonactivating condition (fig. S2B). Spf was also reported to be highly expressed in EHEC under type III secretion permissive conditions in which, as mentioned above, it controls the translation of genes encoded in the EHEC LEE4 operon (*11*).

In total, RIL-seq identified in the two conditions 910 interactions in which the two interacting RNAs are encoded in EPEC core genome (table S3, summary tab). A total of 479 of these interactions were previously revealed using RIL-seq in *E. coli* K-12 (*16*), suggesting that many of EPEC core RNA-RNA interactions are conserved across strains and growth conditions (table S3, summary tab).

#### Novel sRNAs identified in EPEC interactome

We recently showed that sRNAs included in the S-chimeras of Hfq RIL-seq data exhibit particular features, the identification of which may be used to detect novel sRNAs in the data (*16*). These features include the following: (i) sRNAs tend to be the second RNA in the chimeric fragment, (ii) sRNAs have a relatively long U tract at their 3′ ends, and (iii) sRNAs are typically involved in multiple interactions with mRNA regions corresponding to CDS or 5′UTR. Using these features (table S5), we identified in RIL-seq data novel sRNAs encoded in both the core and accessory genome of EPEC (table S6), where we defined accessory genome regions as those mapped to the EPEC plasmids or to prophages and integrative elements listed in table S2 of Iguchi *et al.* (*33*). Several of these novel sRNAs could be further substantiated by the identification of a common motif in their target sequences, which was complementary to the sRNA sequence [seed sequence (*34*)] (fig. S5 and table S7). The orthologs of the novel core-encoded sRNAs are likely to be also expressed in *E. coli* K-12, as they were detected by RIL-seq in K-12 interactome (*16*), but they were not reported since their chimeric fragments in the K-12 strain were too scarce to have them classified as sRNAs. This implies that the repertoire of sRNAs in *E. coli* species has not yet been exploited, and it may further be expanded by RIL-seq analysis of bacteria grown under different conditions and of additional strains belonging to different branches of the *E. coli* phylogenetic tree.

**Fig. 3.**
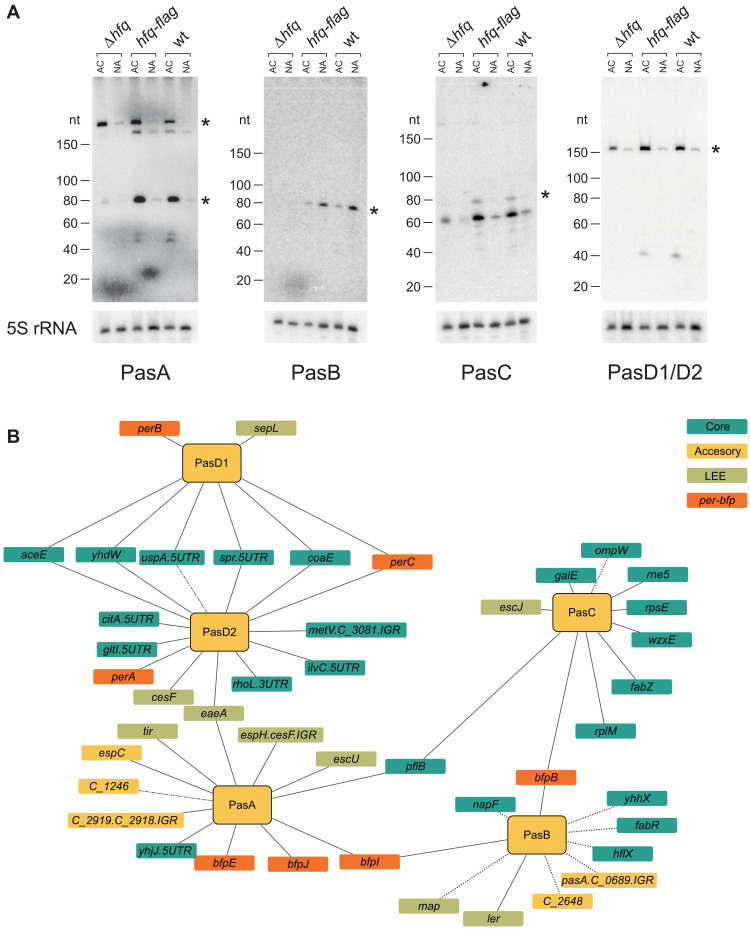
Newly identified sRNAs encoded in genomic islands. (**A**) Northern blot analysis using probes specific to putative sRNAs encoded in the accessory genome (PasA-D1/D2). Total RNA was extracted from three EPEC strains (Δ*hfq*, *hfq-*flag, and wt) grown under activating (AC) or nonactivating (NA) conditions. Asterisks denote the bands with sizes matching those predicted by the RIL-seq and RNA-seq data. Since PasD1 and PasD2 are almost identical (see fig. 6C), we were unable to differentiate them by the use of specific probes, and they are represented by one blot with a single probe. As a loading control, membranes were blotted with a 5S rRNA specific probe. nt, nucleotide. (**B**) RNA-RNA interaction network of the five newly identified sRNAs, all of which are encoded in prophages (table S6). Interactions detected under the activating condition are depicted with solid lines, interactions detected under the nonactivating condition are depicted in dotted lines, and interactions detected under both conditions are depicted in dashed-dotted lines. Turquoise nodes represent transcripts encoded in the core genome. Green, orange, and yellow nodes denote LEE, *per*-*bfp*, and other accessory genome transcripts, respectively. Genes lacking a common name are noted by their locus tag, where C stands for E2348C. Transcripts encoded in intergenic regions (IGR) are denoted as “X.Y.IGR.”

Several novel sRNAs are encoded in the accessory genome (table S6). We selected for experimental verification five of the accessory genome–encoded sRNA candidates, termed here PasA, PasB, PasC, PasD1, and PasD2 (Pas, for “pathogenicity associated small RNA”). These sRNA candidates ranked high by their number of interactions, including interactions with virulence genes (tables S3 and S5), and three of them showed complementarity to a common motif identified in their target RNA sequences (fig. S5). We performed Northern blot analysis that corroborated the sizes of these sRNAs as predicted by the RNA-seq read coverage (80 to 240 nucleotides; fig. S6) and their Hfq-dependent stability (Fig. 3A). PasA, PasC, PasD1, and PasD2 were found to be expressed under the activating condition (Fig. 3A), whereas the expression of PasB was elevated under the nonactivating condition, in accord with their RNA-seq–based expression levels and the numbers of identified interactions (Fig. 3A, table S3, and fig. S2B). While PasA and PasC show partial sequence similarity (fig. S6B), PasD1 and PasD2, encoded within different prophages, are nearly identical, with only four mismatches in their sequences (fig. S6C). We thus could not differentiate between them using Northern blot analysis (Fig. 3A), but the RNA-seq and RIL-seq analyses could partially differentiate between PasD1 and PasD2 and showed their individual expression. These findings support the conjecture that both PasD1 and PasD2 are expressed and interact with target RNAs in an Hfq-dependent manner, and many of these targets are shared (Fig. 3B). The RIL-seq analysis showed that all Pas sRNAs interact with targets encoded in both the core and accessory genome, including T3SS and *bfp* genes (Fig. 3B). Together, our results suggest that at least five of the accessory genome–encoded sRNA candidates identified by RIL-seq are genuine sRNAs with a potential to affect EPEC’s virulence. Another EPEC-specific novel sRNA candidate (locus tag, E2348_P3_01.AS1) originated from the small plasmid p5217 (*35*), which has been reported for only a few EPEC isolates. This putative sRNA was found with the highest number of interacting partners among the accessory genome sRNAs (73 interactions), mostly under the nonactivating condition (tables S3 and S6). Further functional analysis of these newly identified, virulence-associated, sRNAs is left for future work.

#### *Cross-talk between the core and accessory genome* via *sRNA-target interactions*

**Fig. 4.**
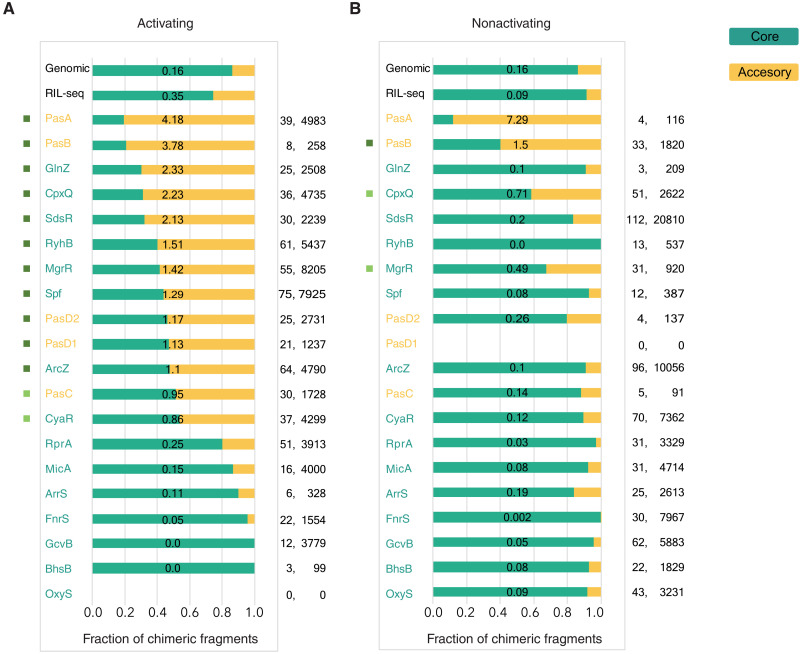
Changes in the repertoire of sRNA targets are associated with differences in growth conditions. For each sRNA in S-chimeras, presented are the fractions of its respective chimeric fragments that include target RNAs originating from core (turquoise) or accessory (yellow) genomic regions, under activating (**A**) and nonactivating (**B**) growth conditions. The ratio between the number of accessory genome–encoded to core-encoded targets is written on the corresponding bar, for each sRNA and condition. Counts are based on table S3. Included are all sRNAs RIL-seq identified to interact with at least 15 different RNA fragments in at least one of the conditions. Chimeric fragments involving sRNA-sRNA interactions were counted per each sRNA. The number of targets and the number of the chimeric fragments are shown to the right. These numbers are not normalized and should not be compared between different sRNAs or different conditions. The core/accessory genome ratio, however, can be compared. The “RIL-seq” category at the top of the panels represents the global fractions of chimeric fragments originating from core and accessory genome regions in all RIL-seq S-chimeras. The “Genomic” category refers to the relative fractions of nucleotides in core and accessory genome regions across the genome. χ^2^ goodness of fit (one sample test) was applied for each sRNA with at least six statistically significant chimeras, testing whether the core/accessory genome distribution of its chimeric fragments deviated statistically significantly from the one expected by the genomic nucleotide distribution. Bonferroni correction for multiple hypotheses was applied separately to the activating and nonactivating condition. Cramers’ V was used to calculate effect size. A colored rectangle to the left of the sRNA designates a statistically significant deviation from the expected distribution with effect size ES > 1 (dark green); 0.5 > ES ≤ 1 (light green).

Examination of the targets of core-encoded and accessory genome–encoded sRNAs revealed that under both conditions, core-encoded sRNAs and accessory genome–encoded sRNAs were found to interact with both core-encoded and accessory genome–encoded targets. Several sRNAs interact mostly with core-encoded targets in one condition and with accessory genome–encoded targets in the other condition (Fig. 4). As many accessory genome–encoded genes are virulence related, the higher the ratio of accessory genome–encoded to core-encoded targets, the more likely it is that the sRNA is involved in virulence regulation. For several sRNAs, such as MgrR, RyhB, and Spf, there is a shift toward accessory genome–encoded targets in the activating condition (Fig. 4), which is associated with an increase in their relative abundances in the activating condition (Fig. 2). Many of these accessory genome–encoded targets correspond to virulence-associated genes. MicA, GcvB, and RprA, which have lower relative fractions of accessory genome–encoded targets, have similar relative fractions of chimeric fragments in the activating and nonactivating conditions (Fig. 4). Furthermore, the majority of their chimeric fragments involve targets that are shared between the activating and nonactivating conditions, although the actual number of targets could differ (fig. S7). This may suggest these sRNAs are not involved in the virulence process.

### Virulence-associated sRNA interactome

**Fig. 5.**
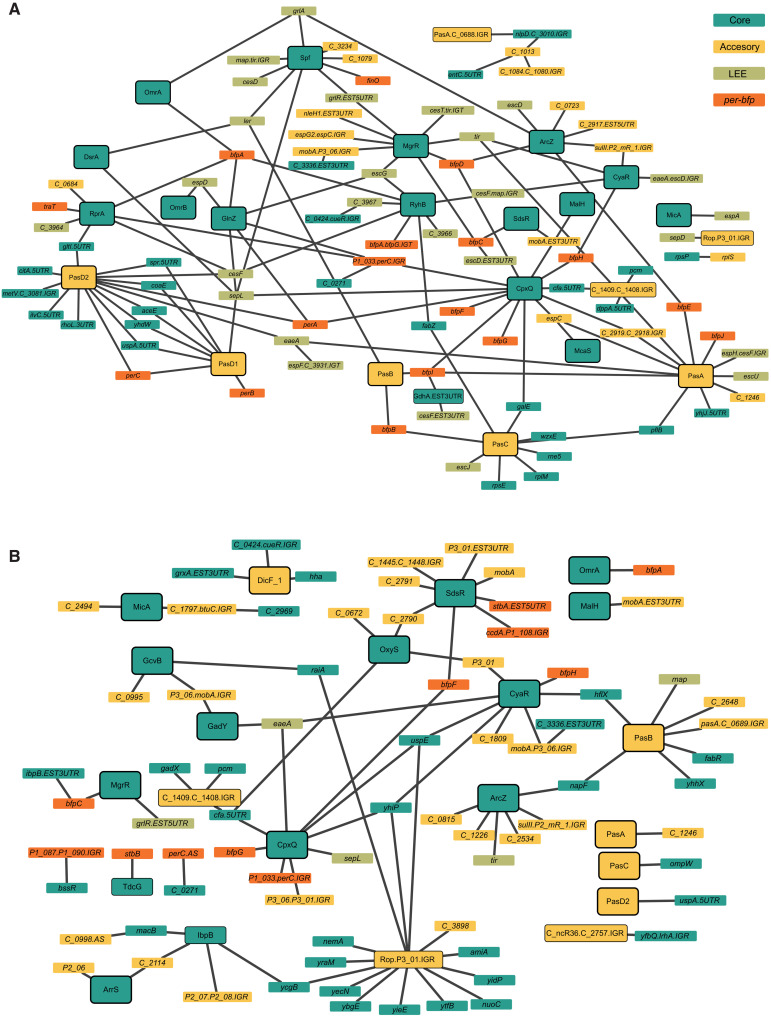
RIL-seq RNA-RNA virulence-associated interaction networks. The virulence-associated RNA-RNA interactions identified by RIL-seq under activating (**A**) and nonactivating (**B**) conditions are represented as networks, where nodes represent RNAs and edges connect nodes for which an interaction was determined. These networks are filtered for interactions detected in at least two of the triplicate libraries, where for each edge, at least one node is an RNA fragment encoded in the accessory genome. Green, orange, and yellow nodes denote LEE, *per*-*bfp*, and other accessory genome transcripts, respectively. Turquoise nodes represent genes encoded in the core genome (Supplementary Material). A node representing a transcript predicted or known to be an sRNA is marked by a black frame.

To obtain a global view of the impact of sRNAs on EPEC virulence, we focused on sRNAs interacting with at least one target that is encoded in the accessory genome of EPEC. To increase the reliability of the results, we considered only RNA-RNA interactions that were identified in at least two individual libraries in addition to their identification in the unified library (*26*). This analysis resulted in two networks, under the activating and nonactivating conditions, in which nodes are sRNAs or targets and edges connect between RNAs identified as interacting by RIL-seq (hereinafter, virulence-associated networks; see Supplementary Methods). The virulence-associated network under the activating condition contained 110 nodes and 136 edges (Fig. 5A), 34 (25%) of the interactions involved *per-bfp* genes, and 45 (33%) involved LEE genes. Under the nonactivating condition, the virulence-associated network is less dense, containing only 97 nodes and 90 edges (Fig. 5B); nine (10%) of the interactions involved *per-bfp* genes and seven (7.8%) involved LEE genes. Notably, several sRNAs, such as MgrR and Spf, are hubs of interactions involving virulence-associated RNAs in the network of activating condition.

### MgrR down-regulates CesT production

**Fig. 6.**
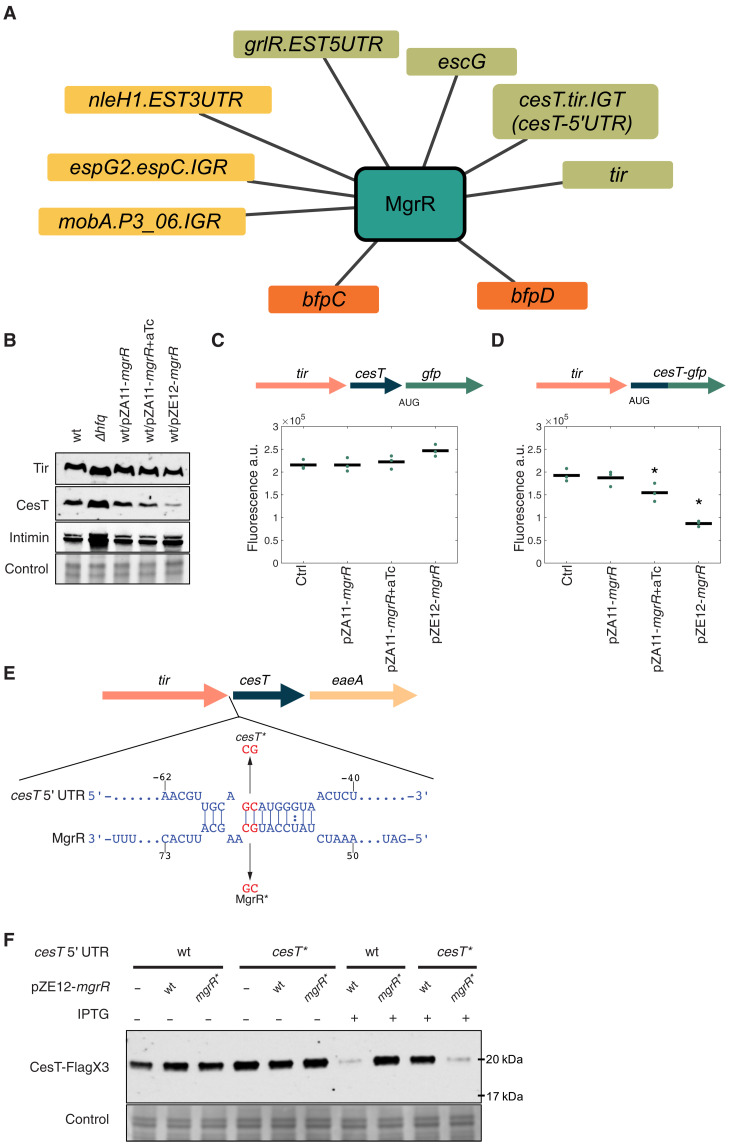
MgrR controls *cesT* expression. (**A**) Network of MgrR interactions with accessory genome–encoded genes, revealed by RIL-seq. Network annotation is as in Fig. 5. (**B**) Western blots of Tir, CesT, and intimin extracted from wild-type EPEC (wt), Δ*hfq* mutant, and EPEC carrying either a plasmid encoding *mgrR* under the inducible Tet promoter (pZA11-*mgrR*) or a plasmid constitutively expressing *mgrR* (pZE12-*mgrR*), grown under the activating condition. Where indicated, anhydrotetracycline (aTc) was used to induce MgrR expression. Total protein was used as a loading control. Representative blots of three independent experiments. (**C** and **D**) EPEC strains containing *cesT-gfp* transcriptional (C) or translational (D) fusion, transformed with either pZA11-*mgrR* or pZE12-*mgrR* plasmids, were grown under activating condition and analyzed for GFP fluorescence intensity. The genetic context of the *cesT*-*gfp* fused gene is displayed above the plot, with different genes represented as arrows. Means of three biological repeats are displayed by black lines, while individual repeats are shown (green dots). Error bars signify SEs. Asterisks denote statistically significantly lower fluorescence compared to the control (*P* ≤ 0.05, Wilcoxon rank sum test, one-tailed). a.u., arbitrary units. (**E**) IntaRNA (*39*) predicted base pairing of *cesT* 5′UTR region and MgrR. In red are mutated nucleotides for the cesT* and MgrR* mutations. LEE5 genes are depicted by arrows. (**F**) Western blots of CesT-3xFlag from wild-type (wt) and cesT* mutant strains, carrying plasmids expressing wild-type MgrR or MgrR* mutant (pZE12-mgrR/mgrR*), grown under the activating condition. Where indicated, isopropyl-β-d-thiogalactopyranoside (IPTG) was added to induce expression of MgrR or MgrR*. Representative blots of three independent experiments.

The virulence-associated network suggests that MgrR functions as a virulence hub, interacting with multiple virulence-associated RNAs (Fig. 6A and table S3). Furthermore, a previous study demonstrated that MgrR regulates the bicistronic *grlRA* transcript, which encodes GrlA, a transcriptional activator of LEE genes, and GrlR, which binds and antagonizes the GrlA activity (*12*). MgrR binds at the 5′UTR of the *grlR* gene, down-regulating GrlR expression and reducing the repression of GrlA by GrlR. As a result, GrlA activity is promoted, leading to up-regulation of transcription of the LEE genes (*12*). The RIL-seq data confirmed the MgrR-*grlR* interaction and further showed that MgrR interacts with eight additional accessory genome–encoded targets, seven of which are virulence associated (Fig. 6A). Of these, the most prominent was MgrR interaction with a sequence located at the *tir-cesT* intergenic region close to the *cesT* translation initiation codon, and thus termed here as *cesT* 5′UTR. This interaction showed the highest number of chimeric fragments involving MgrR and an RNA transcribed from the accessory genome and was identified in all three libraries under the activating condition. *cesT* encodes the CesT protein, which is the major T3SS chaperon and a key posttranscriptional regulator that affects the expression pattern of multiple virulence genes (*36*, *37*), and therefore, we decided to focus our study on this interaction. To find out how MgrR may base pair with the *cesT* 5′UTR, we applied the MEME algorithm (*38*) to the sequences of MgrR targets and identified a common motif, which was found to be complementary to positions 55 to 63 of the MgrR sequence, determining it as the seed sequence of MgrR (fig. S5). Of note, a highly similar motif was identified in the analysis of K-12 MG1655 MgrR RIL-seq targets (*16*). The MgrR binding site, represented by the MEME motif, was identified in the *cesT* 5′UTR, 45 nucleotides upstream to the *cesT* translation initiation codon. The same MgrR binding site in *cesT* mRNA was independently predicted by IntaRNA (*39*).

We then applied Western blot analysis to test the impact of MgrR expression on the levels of the three proteins encoded by the LEE5 operon: Tir, CesT, and intimin. We included an *hfq* mutant in this analysis to confirm that this regulation is Hfq dependent and to assess the consistency of the Western blot results with the RNA-seq results (table S2). Consistent with the RNA-seq results (table S2), the blots showed a slight increase in Tir, CesT, and intimin levels in EPEC Δ*hfq* compared to the wild-type strain (Fig. 6B). Overexpression of MgrR resulted in a dose-dependent decrease in CesT level, whereas the levels of Tir and intimin changed only slightly (Fig. 6B). When comparing wild-type EPEC to a Δ*mgrR* mutant, similar levels of LEE5 proteins were observed in the mutant and wild-type strains (fig. S8A). To characterize the mechanism by which MgrR down-regulates the production of CesT, we fused *gfp* to the chromosomal *cesT*, generating two strains that contain either a transcriptional or a translational fusion. Overexpression of a plasmid-born MgrR in these strains caused a substantial decrease in the fluorescence intensity of the translational fusion, while the transcriptional fusion showed no decrease in green fluorescent protein (GFP) intensity (Fig. 6, C and D), suggesting that MgrR inhibits CesT expression by a posttranscriptional mechanism. This conclusion was further supported by combining Western blot and quantitative polymerase chain reaction analyses, showing that MgrR does not affect *cesT* mRNA level but inhibits CesT posttranscriptionally in a dose-dependent manner (fig. S8, B to D).

To find out whether MgrR interacts with its putative binding site upstream to the translation initiation site of *cesT*, we have mutated two adjacent nucleotides in the identified binding site (Fig. 6E). For simplicity, we termed this mutation cesT*. Of note, the cesT* mutation did not affect the levels of Tir, CesT, and intimin (fig. S8A). Overexpression of MgrR in EPEC cesT* mutant strain could no longer repress CesT production (Fig. 6F). Notably, overexpression of a mutant MgrR that carries the compensatory mutations (termed here as MgrR*) restored *cesT* repression in EPEC cesT* but did not repress *cesT* in wild-type EPEC (Fig. 6F). These findings strongly suggest that MgrR represses CesT production by direct binding at the 5′UTR of *cesT* mRNA.

### Repression of CesT by MgrR enhances EPEC cytotoxicity

**Fig. 7.**
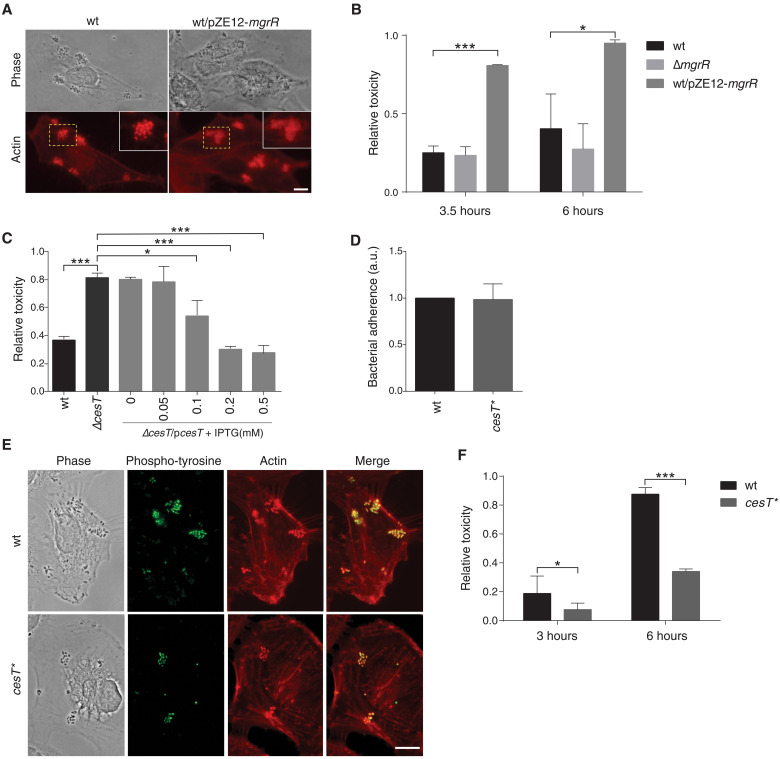
MgrR-*cesT* axis modulates EPEC cytotoxicity. (**A**) Microscopy of HeLa cells infected by wild-type EPEC (wt) or by EPEC overexpressing MgrR (wt/pZE12-*mgrR*) for 2.5 hours stained with phalloidin-rhodamine (actin, red). Yellow dashed squares indicate enlarged areas. Scale bar, 10 μm. (**B**, **C**, and **F**) Toxicity assays (see Supplementary Methods). Different EPEC strains were used to infect human embryonic kidney 293T cells stably expressing GFP. Cytotoxicity was measured at different time points after infection as described (Supplementary Methods). The toxicity relative to noninfected cells is shown. Error bars indicate SD of three biological repeats. Asterisks denote statistically significant differences in toxicity for indicated comparisons (*P ≤ 0.05, ***P ≤ 0.001; two-tailed *t* test). (B) Wild-type EPEC (wt), Δ*mgrR*, or EPEC overexpressing MgrR (wt/pZE12-*mgrR*) were used. Cytotoxicity was measured 3.5 and 6 hours after infection. (C) Wild-type EPEC (wt), EPEC lacking *cesT* (Δ*cesT*), and Δ*cesT* complemented with a plasmid expressing *cesT* under the inducible *tac* promoter (Δ*cesT*/p*cesT*) were used. At 2 hours after infection, IPTG was added to the indicated final concentrations. Cytotoxicity was measured 3.5 hours after infection. (**D**) Bacterial attachment assays of wild-type EPEC (wt) and cesT* mutant. Error bars indicate SD of three biological repeats. (**E**) Microscopy of HeLa cells infected by EPEC wild-type or cesT* mutant for 3 hours. Staining of antiphosphotyrosine used to specifically visualize translocated Tir (green) and with phalloidin-rhodamine to visualize the actin pedestals (red). Scale bar, 10 μm. (F) Toxicity assay of wild-type EPEC (wt) and cesT* mutant. Cytotoxicity was measured at 3 and 6 hours after infection.

We next asked what is the influence of *cesT* repression by MgrR on EPEC virulence, if any. To this end, we first examined the impact of MgrR overexpression on EPEC infectivity using a tissue culture epithelial cell model. Examination of the infected cells 2.5 hours after infection showed that EPEC overexpressing MgrR formed typical BFP-dependent microcolonies on the surface of infected cells, but pedestal formation was partially impaired (Fig. 7A), in line with repression of CesT, known to be required for pedestal formation (*40*, *41*). Unexpectedly, however, EPEC overexpressing MgrR seemed to cause higher cytotoxicity compared with wild-type EPEC. To determine the cytotoxicity level, we used host cell detachment as a proxy for cytotoxicity. To quantify the host cell detachment, we infected GFP-expressing epithelial cells, washed the detached cells, and used a fluorimeter to measure the amount of the GFP that remained associated with the surface of the dish. The results showed enhanced cytotoxicity associated with 3 and 6 hours of infection with the MgrR-overexpressing strain (Fig. 7B). Since MgrR affects many transcripts, we wanted to test whether the higher cytotoxicity is caused by MgrR’s down-regulation of CesT. To test this prediction, we compared the cytotoxicity of wild-type EPEC to that of a mutant lacking *cesT* (Δ*cesT*) and found that the *cesT* deletion mutant is highly toxic to the host cell (Fig. 7C). Furthermore, complementing the Δ*cesT* mutant with a plasmid expressing CesT restored toxicity to its low level in a dose-dependent manner (Fig. 7C). Together, our data show that EPEC lacking CesT is hypertoxic to host cells, suggesting that MgrR overexpression mediates its cytotoxicity via repression of CesT expression.

We then asked whether the level of cytotoxicity associated with *cesT* is affected by the direct interaction of MgrR with the *cesT* 5′UTR. To address this question, we compared the infectivity and cytotoxicity of wild-type EPEC to that of EPEC cesT* mutant, which is not repressed by MgrR. Following 3 hours of infection, we could not detect clear differences in host attachment, formation of microcolonies, Tir translocation, and formation of actin pedestal between the two strains (Fig. 7, D and E). However, the cesT* mutant exhibited reduced cytotoxicity toward the infected host cell, as compared with the wild-type strain (Fig. 7F). These results imply that in wild-type EPEC, the partial CesT repression by the natively expressed MgrR resulted in an increased cytotoxicity. Cumulatively, our results support the notion that natively expressed MgrR sRNA modulates the extent of EPEC cytotoxicity by adjusting the levels of CesT production. How CesT functions to reduce the cytotoxicity of EPEC remains an open question to be addressed in a future study.

## DISCUSSION

In recent years, Hfq was established as a major virulence regulator (*42*–*44*). Comparison of wild-type and Δ*hfq* strains of pathogenic bacteria at the transcriptome, proteome, and functional levels revealed phenotypes associated with virulence as well as changes in the expression levels of virulence-related genes [e.g., (*24*, *45*, *46*) and reviewed in (*42*, *43*)]. Here, we have used RNA-seq–based analyses to interrogate the involvement of Hfq and sRNAs in controlling EPEC virulence. Comparing wild-type and Δ*hfq* strains grown under activating and nonactivating conditions, we found that Hfq functions in regulation of virulence genes. To further explore how Hfq executes its regulation, we deciphered the Hfq-dependent RNA-RNA interactome of EPEC by RIL-seq. We found many K-12 sRNA orthologs in the EPEC interactome, some of which were not annotated in the EPEC genome in public databases. Furthermore, many of the interactions identified previously in *E. coli* K-12 MG1655 (*16*) were rediscovered in the current analysis, suggesting that these interactions are involved in regulation of common physiological conditions encountered by both strains. In addition to orthologs of previously reported sRNAs in K-12, we identified 13 novel EPEC sRNA candidates, five of which are encoded in the core genome and are likely conserved in the *E. coli* species. Eight of the novel sRNAs are encoded within the accessory genome, of which four are found also in EHEC O157:H7 (table S6). The cohort of the newly described accessory genome–encoded sRNAs expands the list of unique sRNAs found in EPEC and the closely related EHEC strains. These include sRNA genes located within prophages identified by prediction followed by Northern blot analysis in EPEC (*47*), or identified by Hfq immunoprecipitation [cross-linking and analysis of cDNA (CRAC)] (*48*) and by RNase E-CLASH analysis in EHEC (*49*). Our findings, together with previous reports, suggest that sRNA genes in the *E. coli* genus include a category of highly conserved core genes, required for basic physiology, and a less conserved pool of sRNA genes, located mostly on mobile elements and involved in adaptation to specific niches.

sRNA-mediated regulation of virulence plays an important role in various Gram-negative and Gram-positive bacterial pathogens. While the outcome of the regulation can differ between species and strains, some common regulatory principles have emerged (*42*–*44*), two of which are highly supported and expanded by our results. First, sRNAs often directly regulate major virulence transcription factors and thus indirectly affect an entire virulence regulon (*43*). Our study supports and expands this notion. We identified sRNAs that interact with one or multiple major virulence regulators and/or with other virulence-associated genes, suggesting these regulatory sRNAs are hubs in the virulence network. The larger the ensemble of virulence-related genes directly regulated by a sRNA, the more likely it is a major virulence hub. A notable example is the PinT sRNA of *Salmonella enterica serovar Typhimurium*, which serves as a “timer” in the transition from invasion to intracellular lifestyle (*50*–*52*). PinT regulates seven T3SS-related genes: four genes encoding key transcriptional regulators *hilA*, *rtsA*, *ssrB*, and the global regulator CRP, and three genes encoding secreted effectors *sopE*, *sopE2*, and *steC* (*50*–*52*). Several sRNAs with at least two direct virulence-associated targets were reported in various bacteria (*43*). This pool is further expanded when targets that regulate cellular physiological processes associated with pathogenicity, such as biofilm formation, are also considered (*43*, *44*). For example, the *Vibrio cholerae* sRNA VqmR down-regulates a toxin (*rtx*), a major regulator of biofilm formation (*vpsT*) and *aphA*, which is a major regulator of both biofilm formation and virulence (*53*, *54*). Other sRNAs reported previously to be involved in pathogenicity were identified as virulence hubs by RIL-seq applied to EPEC. These include Spf, reported previously to directly regulate *hilD*, a T3SS activator in *S. enterica serovar Typhimurium* (*55*), and *sepL* of EHEC, which encodes the T3SS secretion switching protein (*11*). In EHEC, *sepL* is regulated by the major virulence regulator Ler. Both *sepL* and *ler* were identified as targets of Spf in EPEC RIL-seq data, establishing a potential regulatory circuit of the form of feedforward loop. Involvement in feedforward loops has been identified for several other sRNAs in the RIL-seq data. For instance, CpxQ interacts with the mRNA of PerA transcription factor and with multiple *bfp* genes in the PerA regulon. Likewise, in *S. enterica*, PinT was shown to regulate *hilA*, and they both regulate *spoL*, defining a feedforward loop (*51*, *56*). Together, using mixed feedforward loops of a sRNA and a transcription factor to control virulence-associated genes might be a common strategy of pathogenic bacteria.

Second, cross-talk between the core genome and horizontally acquired elements was reported for various species [e.g., *S. enterica* (*57*), EHEC (*49*), and *Staphylococcus aureus* (*58*)]. We find extensive interactions between core-encoded sRNAs and accessory genome–encoded targets and between accessory genome–encoded sRNAs and core-encoded targets. These interactions suggest substantial information flow between the core genome and the genomic islands and plasmids, mediated by sRNAs. In addition, we detect interactions between accessory genome–encoded sRNAs and accessory genome–encoded targets. Some sRNAs seem to interact mainly with core-encoded genes in both conditions, while others seem to markedly shift toward interactions with accessory genome–encoded genes under the activating condition (Fig. 4). As accessory genome–encoded genes have higher potential to be associated with virulence, it further suggests that some sRNAs specialize in virulence regulation and function as virulence hubs.

In EPEC grown under the activating condition, the MgrR sRNA interacts with the largest number of virulence-related mRNAs. Until recently, only three targets of MgrR were identified, *eptB* and *ygdQ* in K-12, encoding a protein involved in lipopolysaccharide modification and a predicted inner membrane protein, respectively (*59*), and *grlR* in EPEC, encoding a regulator of virulence genes (*12*). The previous K-12 RIL-seq data (*16*) confirmed the binding of MgrR to the two core-encoded targets *eptB* and *ygdQ*, while in the current EPEC RIL-seq data, all three were confirmed, along with ~60 additional targets, nine of which are encoded in accessory genomic regions unique to EPEC. Ranking the MgrR targets by the number of chimeric fragments (table S3, summary tab), we find that *cesT* is ranked at the top with 1791 chimeric fragments (cesT.tir.IGT in table S3, summary tab), followed by *eptB*, *bfpC*, *ygdQ*, *tir*, and *grlR* (table S3, summary tab). Among the 13 targets with >100 chimeric fragments, nine are unique to EPEC, including one encoded on the small plasmid p5217 (*35*). These findings reinforce the notion that MgrR evolved in EPEC into a major virulence regulator.

Using Western blot analysis, we found that MgrR overexpression influenced the level of CesT, but not that of Tir. Why Tir production was not affected by MgrR overexpression and what is the outcome of MgrR interaction with the *tir* CDS remain open questions. Nevertheless, our data show that MgrR down-regulates CesT expression and consequently affects the EPEC cytotoxicity. The regulation of *cesT* involves base pairing between MgrR and *cesT* mRNA at its 5′UTR, and mutations that disrupt the base pairing prevent the regulation. When overexpressed, MgrR repressed a *cesT*-*gfp* translational fusion but not a transcriptional fusion, indicating that *cesT* regulation is posttranscriptional. The binding site of MgrR is located 45 nucleotides upstream to the *cesT* initiation codon, ruling out a mechanism of direct interference at the ribosome binding site. Translational repression that involves binding of a sRNA at a site located upstream to the ribosome binding site was demonstrated in several regulatory systems. One such example was provided by the noncanonical binding of Spf at the 5′UTR of *sdhC*, which enables Hfq binding at an ARN repeat sequence located downstream to the Spf binding site, near the ribosome binding site, thus preventing translation initiation complex formation (*60*). Another example is the blocking of a translational enhancer sequence by SgrS binding at the 5′UTR of *manY* (*61*). In this case, the sRNA blocks binding of the S1 ribosomal protein to the enhancer AU-rich sequence, preventing translation enhancement. As the region between the MgrR binding site and the ribosome binding site of *cesT* is AU rich and includes multiple ARN triplets, the two mechanisms described above might be applicable in regulation of *cesT* by MgrR.

Our data show that EPEC lacking CesT is hypertoxic to host cells. This observation was unexpected since this mutant is expected to be deficient in translocation of multiple effectors. How the lack of CesT leads to increased toxicity is not known yet. Comparing the toxic effects of wild-type EPEC and cesT* mutant on infected cells, we found that the mutant strain was less toxic. This result suggests that by modulating CesT expression, MgrR adjusts the cytotoxicity of EPEC. Given that CesT functions as the major T3SS chaperon and as a global posttranscriptional regulator, it is likely that cytotoxicity is only one among other consequences of CesT repression by MgrR. Furthermore, in parallel to modulating CesT translation, MgrR enhances the transcription of all the LEE genes, including that of *cesT*, by repressing production of GrlR, which inhibits LEE gene transcription (*10*). Thus, our data expose additional key layers of the emerging intricate network that controls EPEC virulence [reviewed in (*62*–*65*)] (fig. S9).

In summary, by exploiting RNA-seq and RIL-seq analyses, we demonstrate the influence of Hfq and sRNAs on EPEC’s transcriptome and provide a rich resource of EPEC RNA-RNA interactome, especially of the sRNA regulons. Our data show that some sRNAs function as virulence hubs. Focusing on one of these hubs and one target, MgrR and *cesT*, we demonstrate how MgrR controls the cytotoxicity of the pathogen. The new transcriptome-wide datasets provided here pave the way to reveal and study additional modules involving sRNA-target interactions, which will enhance our understanding of the complex regulation underlying bacterial virulence.

## MATERIALS AND METHODS

Strains, plasmids, and general methods are listed and specified in the Supplementary Methods.

### RNA-seq and RIL-seq analyses

EPEC E2348/69 bacteria were grown under activating or nonactivating condition as described in the Supplementary Methods. RIL-seq was performed as previously described by Melamed *et al.* (*25*), with adaptation of the computational analysis to the EPEC genome and annotation (see Supplementary Methods). For each growth condition, RIL-seq was applied to three biological replicates.

For RNA-seq, bacteria were harvested by centrifugation, the pellet was resuspended in TE buffer, mixed with lysosyme (Sigma-Aldrich) in a final concentration of 0.9 mg/ml, and immediately frozen in liquid nitrogen. The frozen pellet was subjected to two cycles of thaw and freeze at 37°C and in liquid nitrogen, respectively, and RNA was extracted using Tri-Reagent (Sigma-Aldrich). RNA-seq libraries were constructed by the RNAtag-seq method (*66*), with some modifications as described in (*25*). Both RIL-seq and RNA-seq libraries were sequenced by paired-end sequencing using Nextseq 500 Sequencer (Illumina). Reads were mapped to EPEC E2348/69 genome version 19, which includes the chromosome NC_011601.1 and three plasmids NC_011602.1, NC_011603.1, and EU580135 (see Supplementary Methods).

Analysis of the RNA-seq libraries and detection of differentially expressed genes were done by DESeq2 (v 1.28.1) (*18*). DESeq2 was run via an R script (R v. 4.0.2) with the default parameters except for the addition of prefiltering of 1 and the exclusion of the independent filtering.

RIL-seq computational analysis procedure was conducted as detailed in Melamed *et al.* (*25*). RIL-seq v. 0.81 was used for the computational analysis.

### Annotation of EPEC sRNAs encoded in the core genome

As we realized that the annotation of sRNA-encoding genes in EPEC genome was only partial, we extended this annotation, identifying by blastn (*27*) the homologs of K-12 sRNA–encoding genes in EPEC core genome (table S4). We used this annotation in the analysis of EPEC RIL-seq data. sRNAs encoded in K-12 were taken from Hör *et al.* (*28*) and Bar *et al.* (*29*).

### Annotation of RIL-seq data

Following Melamed *et al.* (*16*, *25*), each nucleotide along each of the genomic strands was assigned a specific genomic feature. Nucleotides residing within a defined gene are annotated accordingly, i.e., sRNA, small RNA; tRNA, transfer RNA; rRNA, ribosomal RNA; ncRNA, noncoding RNA that is neither sRNA, tRNA, or rRNA; and CDS, the coding region of a protein-coding gene. Nucleotides residing antisense to a defined gene (and not already defined as genes) are termed AS. Nucleotides residing in untranslated regions between same-operon genes are termed IGT, for intergenic region within operon transcript. Nucleotides residing in an intergenic region between nonoperonic genes (for protein-coding genes, the gene region is expanded to include the UTRs) are termed IGR, for intergenic region. Note the difference between IGR and IGT; while both define nucleotides residing between genes, IGT refers to genes sharing the same operon. Nucleotides residing in the immediate flanking 5′ and 3′ ends of a single gene CDS (or first gene and last gene of an operon) are annotated as UTRs, for untranslated regions. If they are similar to the corresponding 3′/5′UTR in the K-12 MG1655 strain, they are termed 5UTR and 3UTR, respectively; otherwise, they are termed EST5UTR and EST3UTR for estimated UTR, as the UTR is estimated to span a region of 100 nucleotides upstream or downstream the CDS, respectively. For additional details, see the Supplementary Methods.

### Network construction and small-scale experiments

Additional information related to the data annotation, network construction, and small-scale experiments is provided in the Supplementary Methods.

## Notes

View/request a protocol for this paper from *Bio-protocol*.

## References

[1] J. P. Nataro, J. B. Kaper, Diarrheagenic *Escherichia coli*. Clin. Microbiol. Rev. 11, 142–201 (1998). 9457432 10.1128/cmr.11.1.142 PMC121379

[2] A. R. Shenoy, R. C. D. Furniss, P. J. Goddard, A. Clements, Modulation of host cell processes by T3SS effectors. Curr. Top. Microbiol. Immunol. 416, 73–115 (2018). 30178263 10.1007/82_2018_106

[3] A. R. Wong, J. S. Pearson, M. D. Bright, D. Munera, K. S. Robinson, S. F. Lee, G. Frankel, E. L. Hartland, Enteropathogenic and enterohaemorrhagic *Escherichia coli*: Even more subversive elements. Mol. Microbiol. 80, 1420–1438 (2011). 21488979 10.1111/j.1365-2958.2011.07661.x

[4] D. Bieber, S. W. Ramer, C. Y. Wu, W. J. Murray, T. Tobe, R. Fernandez, G. K. Schoolnik, Type IV pili, transient bacterial aggregates, and virulence of enteropathogenic *Escherichia coli*. Science 280, 2114–2118 (1998). 9641917 10.1126/science.280.5372.2114

[5] B. Aroeti, G. Friedman, E. Zlotkin-Rivkin, M. S. Donnenberg, Retraction of enteropathogenic *E. coli* type IV pili promotes efficient host cell colonization, effector translocation and tight junction disruption. Gut Microbes 3, 267–271 (2012). 22572833 10.4161/gmic.19814 PMC3427219

[6] T. Tobe, C. Sasakawa, Role of bundle-forming pilus of enteropathogenic *Escherichia coli* in host cell adherence and in microcolony development. Cell. Microbiol. 3, 579–585 (2001). 11553010 10.1046/j.1462-5822.2001.00136.x

[7] T. K. McDaniel, K. G. Jarvis, M. S. Donnenberg, J. B. Kaper, A genetic locus of enterocyte effacement conserved among diverse enterobacterial pathogens. Proc. Natl. Acad. Sci. U.S.A. 92, 1664–1668 (1995). 7878036 10.1073/pnas.92.5.1664 PMC42580

[8] G. Yerushalmi, Y. Litvak, L. Gur-Arie, I. Rosenshine, Dynamics of expression and maturation of the type III secretion system of enteropathogenic *Escherichia coli*. J. Bacteriol. 196, 2798–2806 (2014). 24837293 10.1128/JB.00069-14 PMC4135678

[9] V. H. Bustamante, M. I. Villalba, V. A. García-Angulo, A. Vázquez, L. C. Martínez, R. Jiménez, J. L. Puente, PerC and GrlA independently regulate Ler expression in enteropathogenic *Escherichia coli*. Mol. Microbiol. 82, 398–415 (2011). 21895790 10.1111/j.1365-2958.2011.07819.x

[10] S. Bhatt, A. N. Edwards, H. T. T. Nguyen, D. Merlin, T. Romeo, D. Kalman, The RNA binding protein CsrA is a pleiotropic regulator of the locus of enterocyte effacement pathogenicity island of enteropathogenic *Escherichia coli*. Infect. Immun. 77, 3552–3568 (2009). 19581394 10.1128/IAI.00418-09 PMC2737987

[11] D. Wang, S. P. McAteer, A. B. Wawszczyk, C. D. Russell, A. Tahoun, A. Elmi, S. L. Cockroft, D. Tollervey, S. Granneman, J. J. Tree, D. L. Gally, An RNA-dependent mechanism for transient expression of bacterial translocation filaments. Nucleic Acids Res. 46, 3366–3381 (2018). 29432565 10.1093/nar/gky096 PMC5909449

[12] S. Bhatt, M. Egan, J. Ramirez, C. Xander, V. Jenkins, S. Muche, J. el-Fenej, J. Palmer, E. Mason, E. Storm, T. Buerkert, Hfq and three Hfq-dependent small regulatory RNAs—MgrR, RyhB and McaS—Coregulate the locus of enterocyte effacement in enteropathogenic*Escherichia coli*. Pathog. Dis. 75, ftw113 (2017). 27956465 10.1093/femspd/ftw113 PMC5827581

[13] A. M. Hansen, J. B. Kaper, Hfq affects the expression of the LEE pathogenicity island in enterohaemorrhagic *Escherichia coli*. Mol. Microbiol. 73, 446–465 (2009). 19570135 10.1111/j.1365-2958.2009.06781.x PMC2770234

[14] E. G. H. Wagner, P. Romby, Small RNAs in bacteria and archaea: Who they are, what they do, and how they do it. Adv. Genet. 90, 133–208 (2015). 26296935 10.1016/bs.adgen.2015.05.001

[15] J. Vogel, B. F. Luisi, Hfq and its constellation of RNA. Nat. Rev. Microbiol. 9, 578–589 (2011). 21760622 10.1038/nrmicro2615 PMC4615618

[16] S. Melamed, A. Peer, R. Faigenbaum-Romm, Y. E. Gatt, N. Reiss, A. Bar, Y. Altuvia, L. Argaman, H. Margalit, Global mapping of small RNA-target interactions in bacteria. Mol. Cell 63, 884–897 (2016). 27588604 10.1016/j.molcel.2016.07.026 PMC5145812

[17] S. L. Vogt, T. L. Raivio, Hfq reduces envelope stress by controlling expression of envelope-localized proteins and protein complexes in enteropathogenic *Escherichia coli*. Mol. Microbiol. 92, 681–697 (2014). 24628810 10.1111/mmi.12581

[18] M. I. Love, W. Huber, S. Anders, Moderated estimation of fold change and dispersion for RNA-seq data with DESeq2. Genome Biol. 15, 550 (2014). 25516281 10.1186/s13059-014-0550-8 PMC4302049

[19] T. A. Lehti, J. Heikkinen, T. K. Korhonen, B. Westerlund-Wikstrom, The response regulator RcsB activates expression of Mat fimbriae in meningitic *Escherichia coli*. J. Bacteriol. 194, 3475–3485 (2012). 22522901 10.1128/JB.06596-11 PMC3434760

[20] T. A. Lehti, P. Bauchart, U. Dobrindt, T. K. Korhonen, B. Westerlund-Wikström, The fimbriae activator MatA switches off motility in *Escherichia coli* by repression of the flagellar master operon flhDC. Microbiology 158, 1444–1455 (2012). 22422754 10.1099/mic.0.056499-0

[21] V. I. Martinez-Santos, A. Medrano-Lopez, Z. Saldana, J. A. Giron, J. L. Puente, Transcriptional regulation of the ecp operon by EcpR, IHF, and H-NS in attaching and effacing *Escherichia coli*. J. Bacteriol. 194, 5020–5033 (2012). 22797761 10.1128/JB.00915-12 PMC3430341

[22] S. Shin, M. P. Castanie-Cornet, J. W. Foster, J. A. Crawford, C. Brinkley, J. B. Kaper, An activator of glutamate decarboxylase genes regulates the expression of enteropathogenic *Escherichia coli* virulence genes through control of the plasmid-encoded regulator, Per. Mol. Microbiol. 41, 1133–1150 (2001). 11555293 10.1046/j.1365-2958.2001.02570.x

[23] C. Nadler, Y. Shifrin, S. Nov, S. Kobi, I. Rosenshine, Characterization of enteropathogenic *Escherichia coli* mutants that fail to disrupt host cell spreading and attachment to substratum. Infect. Immun. 74, 839–849 (2006). 16428726 10.1128/IAI.74.2.839-849.2006 PMC1360345

[24] E. A. Shakhnovich, B. M. Davis, M. K. Waldor, Hfq negatively regulates type III secretion in EHEC and several other pathogens. Mol. Microbiol. 74, 347–363 (2009). 19703108 10.1111/j.1365-2958.2009.06856.x PMC2765575

[25] S. Melamed, R. Faigenbaum-Romm, A. Peer, N. Reiss, O. Shechter, A. Bar, Y. Altuvia, L. Argaman, H. Margalit, Mapping the small RNA interactome in bacteria using RIL-seq. Nat. Protoc. 13, 1–33 (2018). 29215635 10.1038/nprot.2017.115

[26] R. Faigenbaum-Romm, A. Reich, Y. E. Gatt, M. Barsheshet, L. Argaman, H. Margalit, Hierarchy in Hfq chaperon occupancy of small RNA targets plays a major role in their regulation. Cell Rep 30, 3127–3138.e6 (2020). 32130912 10.1016/j.celrep.2020.02.016 PMC7059120

[27] S. F. Altschul, T. L. Madden, A. A. Schäffer, J. Zhang, Z. Zhang, W. Miller, D. J. Lipman, Gapped BLAST and PSI-BLAST: A new generation of protein database search programs. Nucleic Acids Res. 25, 3389–3402 (1997). 9254694 10.1093/nar/25.17.3389 PMC146917

[28] J. Hör, G. Matera, J. Vogel, S. Gottesman, G. Storz, Trans-acting small RNAs and their effects on gene expression in *Escherichia coli* and *Salmonella enterica*. EcoSal Plus 9, 10.1128/ecosalplus.ESP-0030-2019, (2020). 10.1128/ecosalplus.esp-0030-2019 PMC7112153 32213244

[29] A. Bar, L. Argaman, Y. Altuvia, H. Margalit, Prediction of novel bacterial small RNAs from RIL-Seq RNA–RNA interaction data. Front. Microbiol. 12, 635070 (2021). 34093460 10.3389/fmicb.2021.635070 PMC8175672

[30] I. A. Iosub, M. Marchioretto, R. W. van Nues, S. M. Kellar, G. Viero, S. Granneman, The mRNA derived MalH sRNA contributes to alternative carbon source utilization by tuning maltoporin expression in *E. coli*. RNA Biol. 18, 914–931 (2021). 33043783 10.1080/15476286.2020.1827784 PMC8081044

[31] M. Kawano, A. A. Reynolds, J. Miranda-Rios, G. Storz, Detection of 5′- and 3′-UTR-derived small RNAs and cis-encoded antisense RNAs in *Escherichia coli*. Nucleic Acids Res. 33, 1040–1050 (2005). 15718303 10.1093/nar/gki256 PMC549416

[32] Y. Chao, J. Vogel, A 3′ UTR-derived small RNA provides the regulatory noncoding arm of the inner membrane stress response. Mol. Cell 61, 352–363 (2016). 26805574 10.1016/j.molcel.2015.12.023

[33] A. Iguchi, N. R. Thomson, Y. Ogura, D. Saunders, T. Ooka, I. R. Henderson, D. Harris, M. Asadulghani, K. Kurokawa, P. Dean, B. Kenny, M. A. Quail, S. Thurston, G. Dougan, T. Hayashi, J. Parkhill, G. Frankel, Complete genome sequence and comparative genome analysis of enteropathogenic *Escherichia coli* O127:H6 strain E2348/69. J. Bacteriol. 191, 347–354 (2009). 18952797 10.1128/JB.01238-08 PMC2612414

[34] K. Papenfort, M. Bouvier, F. Mika, C. M. Sharma, J. Vogel, Evidence for an autonomous 5′ target recognition domain in an Hfq-associated small RNA. Proc. Natl. Acad. Sci. U.S.A. 107, 20435–20440 (2010). 21059903 10.1073/pnas.1009784107 PMC2996696

[35] C. L. Handford, C. T. Stang, T. L. Raivio, J. J. Dennis, The contribution of small cryptic plasmids to the antibiotic resistance of enteropathogenic *Escherichia coli* E2348/69. Can. J. Microbiol. 55, 1229–1239 (2009). 19940931 10.1139/w09-079

[36] N. Katsowich, N. Elbaz, R. R. Pal, E. Mills, S. Kobi, T. Kahan, I. Rosenshine, Host cell attachment elicits posttranscriptional regulation in infecting enteropathogenic bacteria. Science 355, 735–739 (2017). 28209897 10.1126/science.aah4886

[37] N. Elbaz, Y. Socol, N. Katsowich, I. Rosenshine, Control of type III secretion system effector/chaperone ratio fosters pathogen adaptation to host-adherent lifestyle. mBio 10, e02074-19 (2019). 31530678 10.1128/mBio.02074-19 PMC6751064

[38] T. L. Bailey, C. Elkan, Fitting a mixture model by expectation maximization to discover motifs in biopolymers. Proc. Int. Conf. Intell. Syst. Mol. Biol. 2, 28–36 (1994). 7584402

[39] M. Mann, P. R. Wright, R. Backofen, IntaRNA 2.0: Enhanced and customizable prediction of RNA-RNA interactions. Nucleic Acids Res 45, W435–W439 (2017). 28472523 10.1093/nar/gkx279 PMC5570192

[40] A. Abe, M. de Grado, R. A. Pfuetzner, C. Sánchez-Sanmartín, R. Devinney, J. L. Puente, N. C. Strynadka, B. B. Finlay, Enteropathogenic *Escherichia coli* translocated intimin receptor, Tir, requires a specific chaperone for stable secretion. Mol. Microbiol. 33, 1162–1175 (1999). 10510231 10.1046/j.1365-2958.1999.01558.x

[41] S. J. Elliott, S. W. Hutcheson, M. S. Dubois, J. L. Mellies, L. A. Wainwright, M. Batchelor, G. Frankel, S. Knutton, J. B. Kaper, Identification of CesT, a chaperone for the type III secretion of Tir in enteropathogenic *Escherichia coli*. Mol. Microbiol. 33, 1176–1189 (1999). 10510232 10.1046/j.1365-2958.1999.01559.x

[42] Y. Chao, J. Vogel, The role of Hfq in bacterial pathogens. Curr. Opin. Microbiol. 13, 24–33 (2010). 20080057 10.1016/j.mib.2010.01.001

[43] A. J. Westermann, Regulatory RNAs in virulence and host-microbe interactions. Microbiol. Spectr. 6, 10.1128/microbiolspec.RWR-0002-2017, (2018). 10.1128/microbiolspec.rwr-0002-2017 PMC11633609 30003867

[44] S. Chakravarty, E. Masse, RNA-dependent regulation of virulence in pathogenic bacteria. Front. Cell. Infect. Microbiol. 9, 337 (2019). 31649894 10.3389/fcimb.2019.00337 PMC6794450

[45] A. Sittka, V. Pfeiffer, K. Tedin, J. Vogel, The RNA chaperone Hfq is essential for the virulence of *Salmonella typhimurium*. Mol. Microbiol. 63, 193–217 (2007). 17163975 10.1111/j.1365-2958.2006.05489.x PMC1810395

[46] Y. Pannekoek, R. Huis in ât Veld, C. T. P. Hopman, A. A. J. Langerak, D. Speijer, A. van der Ende, Molecular characterization and identification of proteins regulated by Hfq in *Neisseria meningitidis*. FEMS Microbiol. Lett. 294, 216–224 (2009). 19374669 10.1111/j.1574-6968.2009.01568.x PMC2734931

[47] N. Sudo, A. Soma, A. Muto, S. Iyoda, M. Suh, N. Kurihara, H. Abe, T. Tobe, Y. Ogura, T. Hayashi, K. Kurokawa, M. Ohnishi, Y. Sekine, A novel small regulatory RNA enhances cell motility in enterohemorrhagic *Escherichia coli*. J. Gen. Appl. Microbiol. 60, 44–50 (2014). 24646762 10.2323/jgam.60.44

[48] J. J. Tree, S. Granneman, S. P. McAteer, D. Tollervey, D. L. Gally, Identification of bacteriophage-encoded anti-sRNAs in pathogenic *Escherichia coli*. Mol. Cell 55, 199–213 (2014). 24910100 10.1016/j.molcel.2014.05.006 PMC4104026

[49] S. A. Waters, S. P. McAteer, G. Kudla, I. Pang, N. P. Deshpande, T. G. Amos, K. W. Leong, M. R. Wilkins, R. Strugnell, D. L. Gally, D. Tollervey, J. J. Tree, Small RNA interactome of pathogenic *E. coli* revealed through crosslinking of RNase E. EMBO J. 36, 374–387 (2017). 27836995 10.15252/embj.201694639 PMC5286369

[50] S. Correia Santos, T. Bischler, A. J. Westermann, J. Vogel, MAPS integrates regulation of actin-targeting effector SteC into the virulence control network of Salmonella small RNA PinT. Cell Rep. 34, 108722 (2021). 33535041 10.1016/j.celrep.2021.108722

[51] A. J. Westermann, K. U. Förstner, F. Amman, L. Barquist, Y. Chao, L. N. Schulte, L. Müller, R. Reinhardt, P. F. Stadler, J. Vogel, Dual RNA-seq unveils noncoding RNA functions in host-pathogen interactions. Nature 529, 496–501 (2016). 26789254 10.1038/nature16547

[52] K. Kim, A. D. Palmer, C. K. Vanderpool, J. M. Slauch, The small RNA PinT contributes to PhoP-mediated regulation of the *Salmonella* pathogenicity island 1 type III secretion system in salmonella enterica serovar typhimurium. J. Bacteriol. 201, e00312-19 (2019). 31262841 10.1128/JB.00312-19 PMC6755756

[53] K. Papenfort, K. U. Forstner, J. P. Cong, C. M. Sharma, B. L. Bassler, Differential RNA-seq of *Vibrio cholerae* identifies the VqmR small RNA as a regulator of biofilm formation. Proc. Natl. Acad. Sci. U.S.A. 112, E766–E775 (2015). 25646441 10.1073/pnas.1500203112 PMC4343088

[54] R. Herzog, N. Peschek, K. S. Frohlich, K. Schumacher, K. Papenfort, Three autoinducer molecules act in concert to control virulence gene expression in *Vibrio cholerae*. Nucleic Acids Res. 47, 3171–3183 (2019). 30649554 10.1093/nar/gky1320 PMC6451090

[55] Y. El Mouali, T. Gaviria-Cantin, M. A. Sánchez-Romero, M. Gibert, A. J. Westermann, J. Vogel, C. Balsalobre, CRP-cAMP mediates silencing of *Salmonella* virulence at the post-transcriptional level. PLOS Genet. 14, e1007401 (2018). 29879120 10.1371/journal.pgen.1007401 PMC5991649

[56] I. M. Thijs, S. C. J. De Keersmaecker, A. Fadda, K. Engelen, H. Zhao, M. M. Clelland, K. Marchal, J. Vanderleyden, Delineation of the *Salmonella enterica* serovar Typhimurium HilA regulon through genome-wide location and transcript analysis. J. Bacteriol. 189, 4587–4596 (2007). 17483226 10.1128/JB.00178-07 PMC1913449

[57] V. Pfeiffer, A. Sittka, R. Tomer, K. Tedin, V. Brinkmann, J. Vogel, A small non-coding RNA of the invasion gene island (SPI-1) represses outer membrane protein synthesis from the Salmonella core genome. Mol. Microbiol. 66, 1174–1191 (2007). 17971080 10.1111/j.1365-2958.2007.05991.x

[58] A. Tomasini, K. Moreau, J. Chicher, T. Geissmann, F. Vandenesch, P. Romby, S. Marzi, I. Caldelari, The RNA targetome of *Staphylococcus aureus* non-coding RNA RsaA: impact on cell surface properties and defense mechanisms. Nucleic Acids Res. 45, 6746–6760 (2017). 28379505 10.1093/nar/gkx219 PMC5499838

[59] K. Moon, S. Gottesman, A PhoQ/P-regulated small RNA regulates sensitivity of *Escherichia coli* to antimicrobial peptides. Mol. Microbiol. 74, 1314–1330 (2009). 19889087 10.1111/j.1365-2958.2009.06944.x PMC2841474

[60] G. Desnoyers, E. Masse, Noncanonical repression of translation initiation through small RNA recruitment of the RNA chaperone Hfq. Genes Dev. 26, 726–739 (2012). 22474262 10.1101/gad.182493.111 PMC3323883

[61] M. S. Azam, C. K. Vanderpool, Translation inhibition from a distance: The small RNA SgrS silences a ribosomal protein S1-dependent enhancer. Mol. Microbiol. 114, 391–408 (2020). 32291821 10.1111/mmi.14514 PMC7502529

[62] S. Bhatt, M. Egan, V. Jenkins, S. Muche, J. El-Fenej, The Tip of the Iceberg: On the roles of regulatory small RNAs in the virulence of enterohemorrhagic and enteropathogenic *Escherichia coli*. Front. Cell. Infect. Microbiol. 6, 105 (2016). 27709103 10.3389/fcimb.2016.00105 PMC5030294

[63] R. C. D. Furniss, A. Clements, Regulation of the locus of enterocyte effacement in attaching and effacing pathogens. J. Bacteriol. 200, e00336-17 (2018). 28760850 10.1128/JB.00336-17 PMC5738729

[64] A. Platenkamp, J. L. Mellies, Environment controls LEE regulation in enteropathogenic *Escherichia coli*. Front. Microbiol. 9, 1694 (2018). 30140259 10.3389/fmicb.2018.01694 PMC6094958

[65] A. Serapio-Palacios, B. B. Finlay, Dynamics of expression, secretion and translocation of type III effectors during enteropathogenic *Escherichia coli* infection. Curr. Opin. Microbiol. 54, 67–76 (2020). 32058947 10.1016/j.mib.2019.12.001

[66] A. A. Shishkin, G. Giannoukos, A. Kucukural, D. Ciulla, M. Busby, C. Surka, J. Chen, R. P. Bhattacharyya, R. F. Rudy, M. M. Patel, N. Novod, D. T. Hung, A. Gnirke, M. Garber, M. Guttman, J. Livny, Simultaneous generation of many RNA-seq libraries in a single reaction. Nat. Methods 12, 323–325 (2015). 25730492 10.1038/nmeth.3313 PMC4712044

[67] K. A. Datsenko, B. L. Wanner, One-step inactivation of chromosomal genes in *Escherichia coli* K-12 using PCR products. Proc. Natl. Acad. Sci. U.S.A. 97, 6640–6645 (2000). 10829079 10.1073/pnas.120163297 PMC18686

[68] D. G. Gibson, Enzymatic assembly of overlapping DNA fragments. Methods Enzymol. 498, 349–361 (2011). 21601685 10.1016/B978-0-12-385120-8.00015-2 PMC7149801

[69] Y. Sancak, T. R. Peterson, Y. D. Shaul, R. A. Lindquist, C. C. Thoreen, L. Bar-Peled, D. M. Sabatini, The Rag GTPases bind raptor and mediate amino acid signaling to mTORC1. Science 320, 1496–1501 (2008). 18497260 10.1126/science.1157535 PMC2475333

[70] T. Morita, K. Maki, H. Aiba, RNase E-based ribonucleoprotein complexes: Mechanical basis of mRNA destabilization mediated by bacterial noncoding RNAs. Genes Dev. 19, 2176–2186 (2005). 16166379 10.1101/gad.1330405 PMC1221888

[71] M. Li, I. Rosenshine, H. B. Yu, C. Nadler, E. Mills, C. L. Hew, K. Y. Leung, Identification and characterization of NleI, a new non-LEE-encoded effector of enteropathogenic *Escherichia coli* (EPEC). Microbes Infect. 8, 2890–2898 (2006). 17097322 10.1016/j.micinf.2006.09.006

[72] R. Lutz, H. Bujard, Independent and tight regulation of transcriptional units in *Escherichia coli* via the LacR/O, the TetR/O and AraC/I1-I2 regulatory elements. Nucleic Acids Res. 25, 1203–1210 (1997). 9092630 10.1093/nar/25.6.1203 PMC146584

[73] J. H. Park, K. H. Lee, T. Y. Kim, S. Y. Lee, Metabolic engineering of *Escherichia coli* for the production of L-valine based on transcriptome analysis and in silico gene knockout simulation. Proc. Natl. Acad. Sci. U.S.A. 104, 7797–7802 (2007). 17463081 10.1073/pnas.0702609104 PMC1857225

[74] P. D. Karp, R. Billington, R. Caspi, C. A. Fulcher, M. Latendresse, A. Kothari, I. M. Keseler, M. Krummenacker, P. E. Midford, Q. Ong, W. K. Ong, S. M. Paley, P. Subhraveti, The BioCyc collection of microbial genomes and metabolic pathways. Brief. Bioinform. 20, 1085–1093 (2019). 29447345 10.1093/bib/bbx085 PMC6781571

[75] P. Shannon, A. Markiel, O. Ozier, N. S. Baliga, J. T. Wang, D. Ramage, N. Amin, B. Schwikowski, T. Ideker, Cytoscape: A software environment for integrated models of biomolecular interaction networks. Genome Res. 13, 2498–2504 (2003). 14597658 10.1101/gr.1239303 PMC403769

[76] S. C. Pulvermacher, L. T. Stauffer, G. V. Stauffer, The small RNA GcvB regulates sstT mRNA expression in *Escherichia coli*. J. Bacteriol. 191, 238–248 (2009). 18952787 10.1128/JB.00915-08 PMC2612445

[77] C. M. Sharma, K. Papenfort, S. R. Pernitzsch, H. J. Mollenkopf, J. C. D. Hinton, J. Vogel, Pervasive post-transcriptional control of genes involved in amino acid metabolism by the Hfq-dependent GcvB small RNA. Mol. Microbiol. 81, 1144–1165 (2011). 21696468 10.1111/j.1365-2958.2011.07751.x

[78] T. L. Bailey, M. Gribskov, Combining evidence using p-values: Application to sequence homology searches. Bioinformatics 14, 48–54 (1998). 9520501 10.1093/bioinformatics/14.1.48

