# Supplementary material for: The impact of Hfq-mediated sRNA-mRNA interactome on the virulence of enteropathogenic Escherichia coli
Source: Sci Adv. 2021 Oct 27;7(44):eabi8228. doi: 10.1126/sciadv.abi8228 (PMC8550237; doi:10.1126/sciadv.abi8228)
Supplement: Supplementary file 1 — Supplementary Methods Figures S1 to S9 Tables S9 to S13 Legends for tables S1 to S8 [file sciadv.abi8228_sm.pdf]

Science Advances
AAAS

Supplementary Materials for

The impact of Hfq-mediated sRNA-mRNA interactome on the virulence of enteropathogenic *Escherichia coli*

Sivan Pearl Mizrahi*, Netanel Elbaz, Liron Argaman, Yael Altuvia, Naama Katsowich, Yaakov Socol, Amir Bar, Ilan Rosenshine*, Hanah Margalit*

*Corresponding author. Email: sivanp@mit.edu (S.P.M.); ilanr@ekmd.huji.ac.il (I.R.); hanahm@ekmd.huji.ac.il (H.M.)

Published 27 October 2021, *Sci. Adv.* **7**, eabi8228 (2021)
DOI: 10.1126/sciadv.abi8228

The PDF file includes:

Supplementary Methods
Figures S1 to S9
Tables S9 to S13
Legends for tables S1 to S8

Other Supplementary Material for this manuscript includes the following:

Tables S1 to S8

List of Supplementary Files

Supplementary Table S1 (excel). Number of sequenced fragments in RNA-seq and RIL-seq libraries

Supplementary Table S2 (excel). Differential gene expression analysis (DESeq2)

Supplementary Table S3 (excel). RIL-Seq RNA pairs identified in unified libraries

Supplementary Table S4 (excel). K-12 known sRNAs identified in EPEC

Supplementary Table S5 (excel). Summary of genomic elements identified by RIL-seq

Supplementary Table S6 (excel). Newly identified sRNAs

Supplementary Table S7 (excel). MEME motif alignment

Supplementary Table S8 (text). General Feature Format (GFF) file for EPEC. File generation is described in the Supplementary Methods.

Supplementary Methods

Growth conditions, strains and plasmids

The bacterial strains were constructed using the lambda Red system as described (*67*) and are listed in Supplementary Table S9. Plasmids were constructed using standard methods or isothermal assembly (*68*), and are listed in Supplementary Table S10. Bacteria were routinely grown in Luria-Bertani (LB) medium. Streptomycin (Strp, 50 μg/ml), ampicillin (Amp, 100 μg/ml), or kanamycin (Kan, 40 μg/ml) were added where appropriate. Where indicated, isopropyl-D-thiogalactopyranoside (IPTG, 0.01, 0.05, 0.1, 0.2 and 0.5 mM) or anhydrotetracycline (aTc, 1μg/ml) were added to the growth medium. For the non-activating condition, cultures were grown overnight in LB at 37°C, without shaking. For the activating

condition, the non-activating cultures were diluted 1:100 into Dulbecco's modified Eagle medium (DMEM, Biological Industries) and grown at 37°C without shaking to an OD_{600} of 0.3. Epithelial cells, HeLa, or HEK293, were grown in DMEM supplemented with 10% fetal calf serum (FCS; Biological Industries) and antibiotics (penicillin-streptomycin solution; Biological Industries). A cell line stably expressing GFP was generated with GFP expressing lentivirus as described (*69*).

Supplementary Table S9: Bacterial Strains used in this study

Name	Description	Source
E2348/69	EPEC wild type isolate O127:H6<br>This strain was validated by whole genome sequencing.	J. Kaper
NN5735	E2348/69 Δ*hfq::km*^{R}, termed C1. This strain was validated by whole genome sequencing. This stock has a mix of wt and mutated *perA* (see Supplementary Fig. S1).	This study
NN5734	E2348/69 Δ*hfq::km*^{R}, termed C2. This strain was validated by whole genome sequencing (see Supplementary Fig. S1).	This study
NE9430	E2348/69 Δ*hfq::km*^{R} NN5735 C1 derivative, termed C1a.<br>This strain was validated by whole genome sequencing. It carries the mutated *perA*.	This study
NE9431	E2348/69 Δ*hfq::km*^{R} NN5735 C1 derivative, termed C1b.<br>This strain was validated by whole genome sequencing.	This study
NE9432	E2348/69 Δ*hfq::km*^{R} NN5735 C1 derivative, termed C1c.<br>This strain was validated by whole genome sequencing. It carries the mutated *perA*.	This study
NE9433	E2348/69 Δ*hfq::km*^{R} NN5735 C1 derivative, termed C1d.<br>This strain was validated by whole genome sequencing. It carries the mutated *perA*.	This study
NE9434	E2348/69 Δ*hfq::km*^{R} NN5734 C2 derivative, termed C2a.<br>This strain was validated by whole genome sequencing. It has lost pMar2.	This study
NE9435	E2348/69 Δ*hfq::km*^{R} NN5734 C2 derivative, termed C2b.<br>This strain was validated by whole genome sequencing. It has lost pMar2.	This study
NE9436	E2348/69 Δ*hfq::km*^{R} NN5734 C2 derivative, termed C2c.<br>This strain was validated by whole genome sequencing. It has lost pMar2.	This study
S-112	E2348/69 Δ*hfq*; constructed from NE9430 by *km*^{R} excision using FLP recombinase (*67*). This is the strain used for RNA-seq. It carries the mutated *perA*.	This study
S-102	E2348/69 *hfq-Flag*; constructed by inserting the *hfq-Flag:cat* fragment from strain TM615 (*70*) followed by *cat* excision using FLP recombinase (*67*). This is the strain used for RIL-seq.	This study
NE7557	E2348/69 containing *cesT-gfp* translational fusion	Elbaz *et al.* (*37*)
NE7558	E2348/69 containing *cesT-gfp* transcriptional fusion	Elbaz *et al.* (*37*)
NE8878	E2348/69 containing *cesT-flagx3* translational fusion; constructed by lambda red knockout of *tir-cesT* intergenic region (IGR) and *cesT*	This study

	CDS using *tetA-sacB* cassette, followed by a pop-out of the latter using a *tir-cesT* IGR and *cesT* CDS:*flagx3* insert created by PCR and ligation.	
NE8810	E2348/69 *cesT**; constructed by lambda red knockout of *tir-cesT* IGR and *cesT* CDS using *tetA-sacB* cassette, followed by a pop-out of the latter using a mutated *tir-cesT* IGR and *cesT* CDS insert created by PCR and ligation.	This study
NE8895	E2348/69 *cesT**, containing *cesT-flagX3* translational fusion; *cesT** mutation was introduced into *cesT-flagx3* containing strain using the lambda red system.	This study
EM2018	E2348/69 Δ*cesT*	Li *et al.* (*71*)
NE9199	E2348/69 Δ*mgrR*; constructed by creating a *mgrR::km*^{R} knockout with lambda red recombination system(*67*)	This study
NE9201	E2348/69 *cesT** Δ*mgrR*; constructed by creating a *mgrR::km*^{R} knockout on the background of *cesT** with lambda red recombination system (*67*)	This study

Supplementary Table S10: Plasmids used in this study

Name	Serial number in our collection	Description	Reference or source
pKD46	p811	Contains the *λ red* genes.	Datsenko *et al* (*67*)
pZA11-MgrR	p7879	*mgrR* under $P_{LtetO-1}$ inducible promoter. p15A origin of replication	This study[a]
pZE12-$MgrR_{K12}$	p7878	*mgrR* was amplified from *E. coli* K12 and cloned under $P_{LlacO-1}$ constitutive promoter. ColEI origin of replication.	This study[a]
pZE12-$MgrR_{EPEC}$	P8980	*mgrR* was amplified from EPEC E2348/69 and cloned under $P_{LlacO-1}$ constitutive promoter. ColEI origin of replication.	This study[a]
pZE12-MgrR*	p8981	*mgrR** under $P_{LlacO-1}$ constitutive promoter. ColEI origin of replication.	This study[a]
pLacI (pREP4)	p2683	*lacI* under its native promoter, p15A origin of replication.	Qiagen.
p-CesT	p6236	*Km*^{R} derivative of the pSA10-*cesT* plasmid,	Katsowich *et al* (*36*)
pZS*-Hfq	p9188	Hfq under its native promoter. pSC101* origin of replication	Shoshy Altuvia's lab
p-mCherry	P2689	mCherry under the Ptac promoter. *lacI*^{q}. ColE1 origin of replication.	Lab collection

[a] constructed using the pZE system (*72*).

Supplementary Table S11. Oligonucleotides used to construct strains and plasmids.

Oligo No.	sequence	Usage
1643	5'-GCATATAAGGAAAAGAGAGAATGGCTAAGGGGCAATCTTTACAAGATCCGGT GTAGGCTGGAGCTGCTTC-3'	Construction of E2348/69 Δ*hfq*
1644	5'- TAAAAAAACAGCCCGAAACCTTATTCGGTTTCTTCGCTGTCCTGTTGCGCCAT ATGAATATCCTCCTTAG-3'	

3317<br>4243<br>4244<br>3405	5'-GCAAGAAGCA TTCAAGAACCCTGAG-3'<br>5'-TGCAACGTTGTTCTCTGAAACATTAAC -3'<br>5'-CAGAGAACAACGTTGCACGATGGGTAACTCTTGAACTTCTGTTATTATAAATC-3'<br>5'- CACTACTTTTGAAATAGTCTCGCCAG -3'	Construction of E2348/69 *cesT**
3876<br>4436<br>5546<br><br>5547	5'- GCAGAAGACGCTTCTCTGAATA-3'<br>5'-TCTTCCGGCGTAATAATGTTTATTATCG-3'<br>5'-GCTCAAGCGATAATAAACATTATTACGCCGGAAGAGACTACAAAGACCATGAC GGTGATTATAAA G -3'<br>5'- CTCCACCACAATGAGTTAGAATGAGTAGTAAATTAATACTAATAAATAAGTTT TACTATTTATCGTCGTCATCTTTGTAG-3'	Construction of *cesT-flagx3*
314<br>315	5’-P- GATTCGTTATCAGTGCAGGAAAAT-3’<br>5’- GTTTTTTTCTAGA GAGATAATCCCTCACCTAACCG-3’	Construction of pZE-MgrR $_{K12}$
5635<br>5636	5'-GGAT AACAAGATACTGAGCACGATCCGTTATCAGTGCAGGAAAATGCCTG-3'<br>5'-ATCGTGCTCAGTATCTTGTTATCCGCT C-3'	Construction of pZE-MgrR $_{EPEC}$
4212<br>4245	5'- ATAGATTTGTGTTTTGCTTTTACGCTAAC-3'<br>5'- GTTAGCGTAAAAGCAAAACACAAATCTATCCATCGAAGCATTCACCGCCGG-3'	Construction of pZE-MgrR*
703<br><br>5593	5'-TTTAAGCTCCGTTTAACATTCATTGAGAAAACTGATGCTACTGT GTCAACATTCCGGGGATCCGTCGACC-3'<br>5'-AAGGGGCCTGATTTTTATGACGGCGAAAAAAAACCGCCAGTAAACCGGCGGTG AAG TGTAGGCTGGAGCTGCTTC-3'	Construction of E2348/69 Δ*mgrR*

Northern blots

For RNA extraction, cultures in the indicated conditions were centrifuged at 4°C and the pelleted cells were resuspended in 50µl 10 mM Tris-HCl (pH 7.5) containing 1 mM EDTA. Lysosyme was added to 0.5 mg/ml, and samples were subjected to three freeze-thaw cycles. RNA was extracted using TRI Reagent (Sigma) according to manufacturer instructions. RNA samples (10µg) were denatured for 10 min at 70°C in 65% formamide, separated on 7 M urea, 6% polyacrylamide gels in 44.5 M Tris-base, 44.5 M Boric acid and 2 mM EDTA pH 8.0, and transferred to Zeta-Probe membrane (BioRad) by electroblotting. The membranes were hybridized with specific [^{32}P] end labelled DNA probes. The probe sequences are listed in Supplementary Table S6.

RNA extraction and real time PCR

Total RNA was extracted using TRI reagent (Sigma) according to the manufacturer's instructions. RNA (1.5 µg) was treated with RQ1 DNase I (Promega) at a concentration of 1U/µg RNA for 30 min at 37°C. DNase I was inactivated by adding 1µl of stop solution and heating the samples for 15 min at 65°C. DNA digestion was verified by PCR, using primers #1952 and #1953. cDNA was synthesized using a qPCRBIO high-quality cDNA synthesis kit (PCR Biosystems) and quantified by Real-Time PCR using iTaq Universal SYBR Green Supermix (Bio-Rad) with a CFX96 Real-Time System (Bio-Rad) according to the manufacturer's instructions. The level of 16S rRNA (*rrsB*) was used to normalize the expression data for *mgrR* and *cesT*. The relative amount of cDNA was calculated by the standard curve method (ΔΔCq), which was obtained by PCR of serially diluted genomic DNA as standard and analyzed using Bio-Rad CFX maestro software.

Supplementary Table S12. Oligonucleotides used for real time PCR

Oligo No.	Oligo name	sequence	Reference or source
1952	*rssB* Fwd	5'-CAGAGATGAGAATGTGCCTTCGGG-3'	Park *et al* (*73*)
1953	*rssB* Rev	5'-CCGCTGGCAACAAAGGATAAGG-3'	Park *et al* (*73*)
3863	*cesT* Fwd	5'-TGATTTATGGCGTCTGTGGA-3'	Elbaz *et al* (*37*)
3864	*cesT* Rev	5'-TTTCAGGGGTAGCATCATCG-3'	Elbaz *et al* (*37*)
5640	*mgrR* Fwd	5'-GATCCGTTATCAGTGCAGGAAAATG-3'	This study
5641	*mgrR* Rev	5'-AGTAAACCGGCGGTGAATG-3'	Moon *et al* (*59*)

Western blots

Bacterial cultures OD_{600} was measured and similar amounts of cells were precipitated, resuspended in 1X Laemmli sample buffer (Bio-Rad) and boiled for ten minutes. The extracts were analyzed by western analysis, using the primary antibodies that are listed in Table S12. The loading amounts were further normalized by the total protein amount as recorded from stain-free gels imaging (Bio-Rad) or by Coomassie or Ponceau red staining.

Supplementary Table S13: Antibodies used in this study

Antibody	Source	Dilution
Anti-CesT-Tir	James Kaper	1:1000
Anti-intimin	Gad Frankel	1:2500
Peroxidase AffiniPure Goat Anti-Rabbit IgG (H+L)	Jackson 111-035-003	1:10000
Phospho-tyrosine	P4110 Sigma	1:500
Anti-Flag	Sigma-Aldrich F1804	1:1000 and According to RIL-seq protocol {Melamed, 2018 #71}
Anti BfpA	Gad Frankel	1:5000

Determination of GFP fluorescence intensity

Bacteria were grown in DMEM as indicated above. The cultures were then washed and suspended in phosphate buffered saline (PBS). Then, fluorescence intensity of the GFP was measured (485-nm excitation and 510-nm emission) using a Spark 10M microplate reader (Tecan) and normalized according to the optical density (OD_{600}). Each experiment was performed in triplicates, and the mean value of their normalized fluorescence intensity was calculated.

Infections and microscopy analysis

HeLa cells were seeded in a 24-well plates (Nunc) at a density of $7x10^4$ cells per well and grown overnight in DMEM supplemented with 10% fetal calf serum (FCS; Biological Industries) and antibiotics (penicillin-streptomycin solution; Biological Industries). Next, DMEM was replaced with a fresh DMEM lacking FCS and antibiotics and the HeLa cells were infected with bacteria that had been statically grown overnight at 37°C, at multiplicity of infection of 1:100. The cells and infecting bacteria were incubated for 2.5h or 3h at 37°C in 5% CO_2. The cells were then fixed in 3.7% formaldehyde in PBS for 10 min at room temperature, washed with PBS, perforated with 0.25% Triton X-100 in PBS for 10 min at room temperature, washed with PBS and stained with phospho-tyrosine (visualization of the

translocated Tir protein; green) or phalloidin-rhodamine (visualization of actin clustering/pedestal formation; red) (Sigma). Stained cells were analyzed by fluorescence microscopy using the ZEN software.

Toxicity assay:

HEK293-T stably expressing GFP were seeded in 10cm plates (Nunc) at a density of $5x10^6$ cells per plate and grown overnight in DMEM medium supplemented with 10% fetal calf serum (FCS; Biological Industries) and antibiotics (penicillin-streptomycin solution; Biological Industries) at 37°C in 5% CO2 incubator. For infection, DMEM was replaced with fresh DMEM + FCS lacking antibiotics and cells were infected with bacteria that had been statically grown overnight at 37°C (multiplicity of infection, 1:100), for the indicated times. Where indicated, IPTG was added to induce CesT expression. To terminate the infection cells were washed with PBSx1, the remaining attached cells were scraped and GFP intensity was measured by fluorimetry using Spark 10M microplate reader (Tecan). The toxicity relative to non-infected cells (NI) was calculated as $1-\frac{\text{fluorescence level (infected cells)}}{\text{fluorescence level (NI)}}$.

Bacterial Attachment assay:

HEK-293T cells stably expressing GFP were infected by bacteria cells. At 3 h post infection the infected cells were washed from unattached bacteria and detached host cells and the levels of remaining mCherry (bacteria) and GFP (cells) were measured. The mCherry/GFP ratio was used as readout for bacterial attachment.

Genome annotation

RIL-seq exploits genome annotation of genomic features at a nucleotide resolution (*16, 25*). For EPEC annotation, genes were initially taken from the gene.dat files of the EPEC E2348/69 genome version 19 and the three plasmids it contains: NC_011601.1,

NC_011602.1, NC_011603.1 and EU580135 (taken from NCBI). To these we have added the sRNAs we identified to be homologous to the ones known in *E. coli* K-12 MG1655 (*28, 29*). For these added sRNAs, gene name was adapted from the K-12 MG1655 orthologue (Supplementary Table S4). Also added are the newly identified Pas sRNAs. (The Pas sRNAs were discovered in a run of RIL-seq in which they were not included specifically, but as intergenic or antisense features. They were then verified by northern blot analysis and their genomic location deduced from the northern blot and RNA-seq coverage. All newly sRNA genes were given locus tags as E2348CN_XXXX, where C stands for Chromosome, N for new, and XXXX is a running number. Interacting fragments were classified into the following categories based on their mapping annotation: 5UTR (5'UTR/EST5UTR), Coding Sequence (CDS), 3UTR (3'UTR/EST3UTR), tRNA, sRNA, ncRNA (non-coding RNA), AS (AntiSense), IGR (InterGenic Region) and IGT (InterGenic within operon Transcript). rRNA-derived fragments were filtered out. For each transcription unit (as identified by the transunit.dat file in BioCyc (*74*)), the first and last genes were identified and 5'/3'UTR regions were assigned as follows: If there was a homologous UTR from K-12 MG1655, its annotation was used (named 5/3UTR, respectively), otherwise 100 nucleotides upstream the AUG (or less if there was a gene end in this range) were designated as the 5'UTR (named EST5UTR, for estimated 5'UTR). Similarly, 100 nucleotides downstream the stop codon were designated as the 3'UTR (named EST3UTR). The GFF file that includes all genes used for RIL-seq data analysis can be found in Supplementary Table S8.

For the RNA-seq analysis, we were interested in the differential expression of the individual genes, and therefore only considered reads that were mapped to genes. The annotations were extracted accordingly from the above described GFF file (Supplementary Table S8) to generate the GFF file that was used for the RNA-seq analysis. It is of note that in

the RNA-seq data analysis, reads with ambiguous mapping, overlapping two adjacent annotations, were excluded.

The GFF file was constructed in-house for EPEC E2348/69 genome based on the BioCyC version 19.0 (*74*) and two of the plasmids it contains: NC_011601.1 (chromosome), NC_011602.1, NC_011603.1. Genes encoded on the smaller plasmid EU580135 were inserted manually. New sRNAs were also inserted as explained above.

Each line in the file contains the following information:

1. seqid: genomic element (chromosome or plasmid identification reference);
2. Source: how it was created (parsing of BioCyc version 19.0 (*74*) genes.dat or manually added);
3. Type: RNA classification as one of the following: CDS, ncRNA, tRNA, rRNA, sRNA
4. Start position: coordinates are given in positive 1-based integer
5. End position: coordinates are given in positive 1-based integer
6. Score: numeric value that generally indicates the confidence of the source in the annotated feature. A value of "." (a dot) is used to define a null value.
7. Strand: + for positive strand; – for minus strand.
8. Attributes: A list of feature attributes in the format tag=value. Multiple tag=value pairs are separated by semicolons. locus_tag is a mandatory attribute and serves as a unique key in our analysis.

Generation of network images

The network images were generated using Cystoscape (*75*). The networks include the interactions listed in the summary sheet of Supplementary Table S3. We included in the networks only interactions (edges) that passed the statistical filter in at least two of the triplicate

libraries of the indicated condition (activating/non-activating condition). Self-edges were removed.

To get only the virulence-associated interactions, we have selected all nodes representing RNAs mapped to accessory genome regions. We define accessory genome regions as those residing on plasmids, prophages and integrative elements listed in Table S2 of Iguchi *et al* (*33*). Then, the fragments found to be interacting with these RNAs according to RIL-Seq were added to the selection. All interactions identified for this set of nodes were included in the virulence-associated networks. Note that this selection means that an interaction of two "core" nodes may be included, as long as each of them also interacts with an accessory genome node.

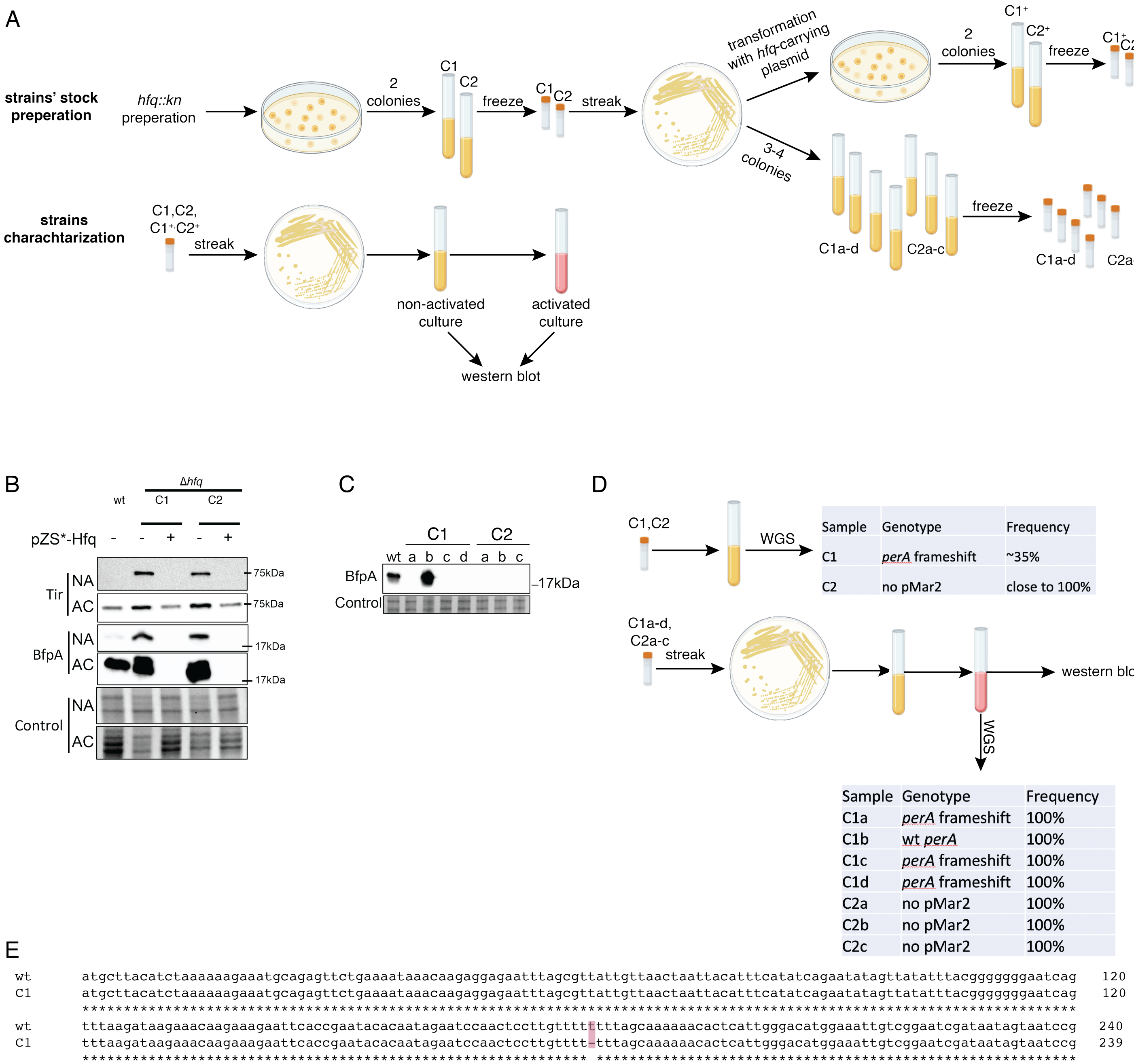

Fig. S1: EPEC *hfq* deletion mutant exhibits unstable BFP expression

<u>**(A)** The flow of construction and analysis of the *hfq* mutants</u>

Upper panel: Two independent *hfq* deletion mutants, designated C1, C2, were constructed and deposited to frozen stocks (indicated by red-cup tubes). Bacteria from the frozen stocks were streaked on a plate and a colony of each mutant was transformed with a plasmid expressing *hfq* (pZS**hfq*) to create strains C1+, C2+ (upward arrow). Colonies derived from C1 and C2

mutants, designated C1a-d and C2a-c, were further cultured in LB (downward arrow). All derivatives of C1 and C2 were deposited to frozen stocks.

Lower panel: For analysis, bacteria from the frozen stokes were streaked and colonies were inoculated to LB, followed by sub-culturing in DMEM to obtain cultures grown under non-activating and activating conditions, respectively.

(**B-C**) Analysis of *hfq* mutants and derivatives by western blots

(**B**) The levels of two representative proteins in the bacteria were tested: Tir, the major T3SS effector, and BfpA, the pilin subunit of BFP. Tir and BfpA levels were compared between wild type, *hfq* mutants (C1, C2) and respective complemented mutants (C1+, C2+). Total protein extracts from cultures grown under non-activating (NA) and activating (AC) conditions were subjected to western blot analysis with anti-Tir and anti-BfpA antibodies. As loading control (Control) we used total protein separated on SDS-PAGE (Bio-Rad stain free gel). Molecular size markers are shown in the right-hand side. The results show that in wild-type EPEC the expression of both Tir and BfpA is higher under the activating condition as compared to the non-activating condition. In the *hfq* mutants the levels of Tir and BfpA are increased in cultures grown under both conditions. Upon *hfq* complementation, the expression levels of Tir were restored to the level seen in the wild type bacteria. In contrast, BfpA amount in the complemented strains dropped below detection levels. Given that BfpA overexpression was shown to cause an envelope stress (*17*), our results hint that during the construction of the complemented strains suppressor mutants that eliminate BfpA expression become dominant in the culture. (**C**) To test if suppressor mutations arise during growth of the *hfq* mutants we have repeated BfpA analysis using several isolated colonies derived from the C1 and C2 cultures (C1a-d and C2a-c, see (A)). Indeed, six of the seven tested colonies have lost BfpA expression, reinforcing the premise that the *hfq* mutant has acquired suppressor mutations that eliminated BfpA expression.

(**D-E**) Whole genome sequencing (WGS) of *hfq* mutants

To assess whether the tested derivates of the *hfq* mutants contain suppressor mutations, we have extracted DNA from the cultures and subjected it to whole genome sequencing (WGS). The steps until DNA extraction for each of the strains used are shown. Tables detail the genotype differences compared to the wild type and their frequency in each sample, according to the WGS analysis. The WGS revealed two types of suppressor mutations:

(*i*) A frameshift mutation in the *perA* gene that encodes the positive regulator of BFP. Sequence alignment of *perA* from wt and Δ*hfq* C1 mutant, shown in (E), indicates a frameshift mutation in the *perA* gene due to deletion of one nucleotide within a stretch of eight T residues, (highlighted in pink). This mutation was present in 35% of the reads while the other 65% showed the wild type sequence, indicating C1 is a mixed culture containing the wild type and a suppressor variant. Analysis of four isolated colonies derived from the C1 mutant (C1a-d) showed that one exhibits wild type genotype (C1b), while all the others contain only the mutated *perA*. This result is in agreement with the blot shown in (C).

(*ii*) Curing of the pMAR2 plasmid that contain all the BFP genes as well as the *perABC* operon involved in BFP regulation. The C2 mutant had very few reads mapped to pMAR2 and DNA extracted from all three derivatives, C2a-c, completely lacked pMAR2 sequences.

Taken together, our results reinforce previous reports suggesting that Hfq functions to repress BfpA expression (*17*). Furthermore, our data suggest that BfpA overexpression due to lack of Hfq is unfavorable in EPEC, possibly by inducing envelope stress (*17*), leading to rapid accumulation of secondary events that eliminate BFP expression altogether.

Thus, to avoid possible variabilities stemming from loss of BFP expression we have used a derivative of C1 strain, in which we removed Km^R, as our Δ*hfq* strain for transcriptome analysis.

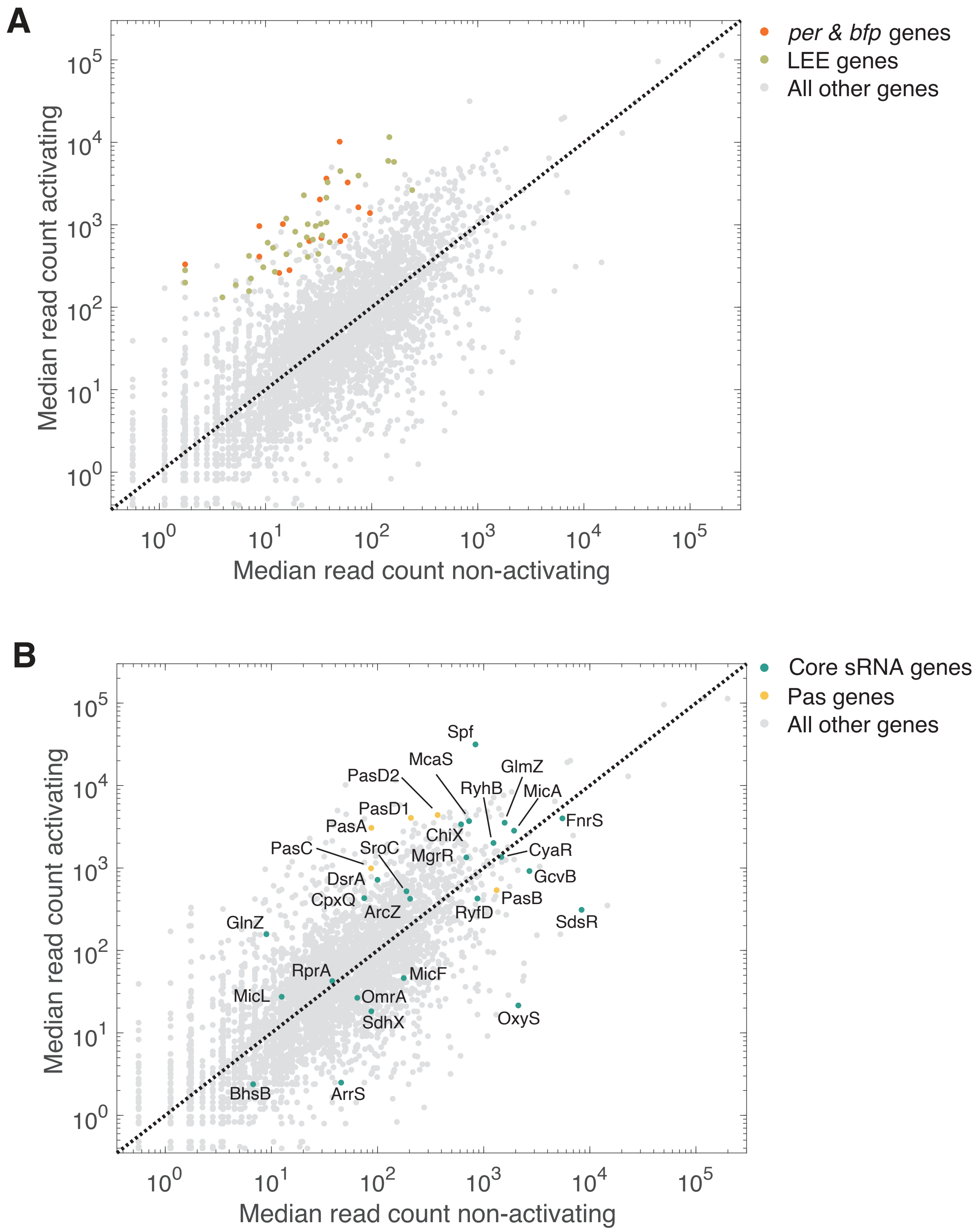

Fig. S2: Comparison of the transcriptomes of EPEC under activating and non-activating conditions. For each gene the median DEseq2 normalized read count of three EPEC wild-type libraries is indicated in activating vs. non-activating conditions. **(A)** *per-bfp* and LEE-related genes are indicated by orange and green dots, respectively. Grey dots represent the rest of the genes. (**B**) Core sRNAs and Pas genes are indicated by turquoise and yellow dots, respectively. Grey dots represent the rest of the genes.

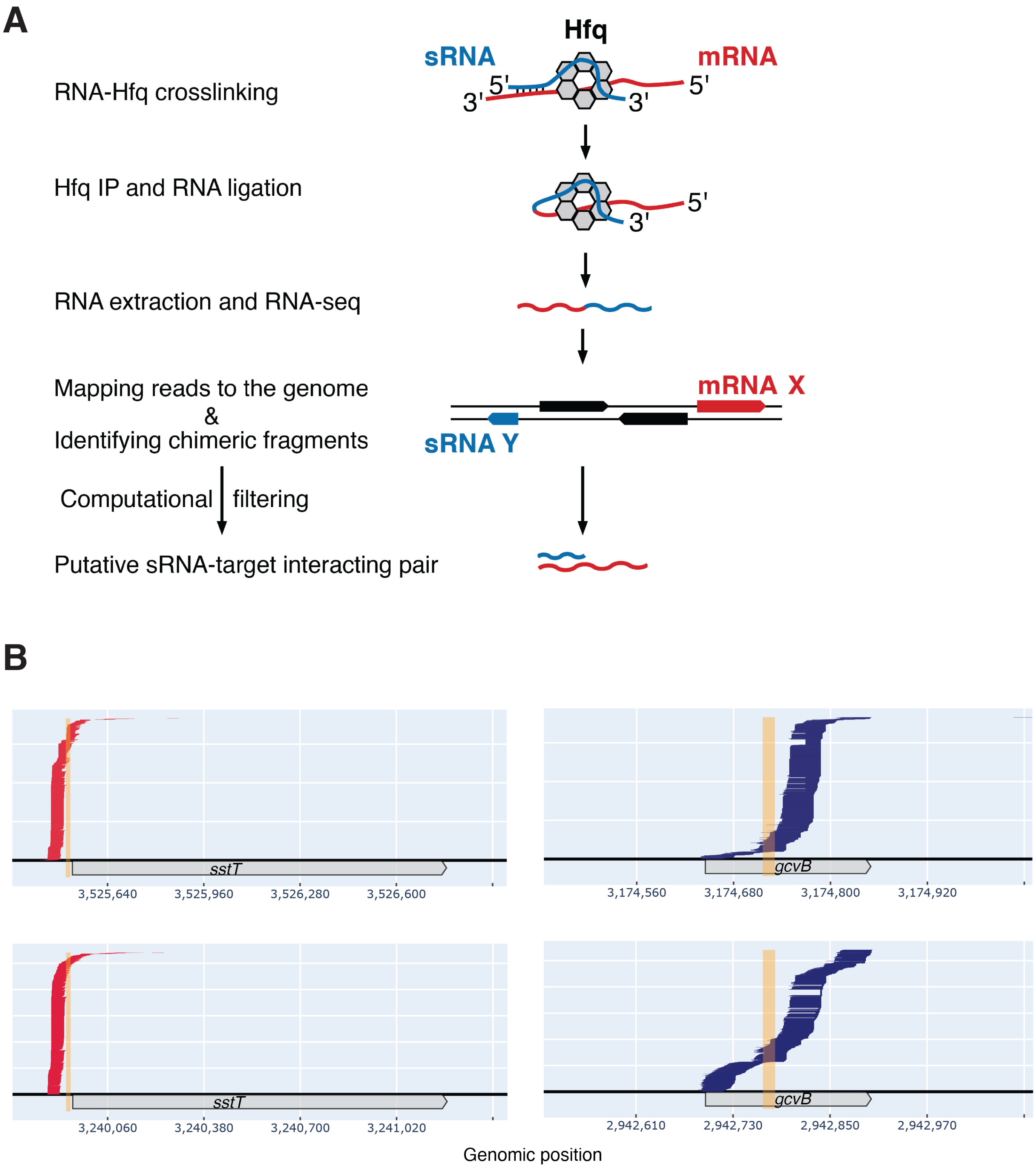

Fig. S3: RIL-seq enables in vivo global mapping of the small RNA interactome
(A) Overview of RIL-seq experimental and computational procedures. (**B**) sstT.5UTR-gcvB is presented as a representative sRNA-target interaction (*76*). Shown are read coverage plots of sstT and GcvB (red and blue lines, respectively) in S-chimeras of the sstT.5UTR-gcvB interaction. Upper panels: data from RIL-seq experiment of EPEC under the non-activating condition. Bottom panels: data from RIL-seq experiment of *E. coli* K-12 grown to stationary phase (*16*). The binding sites on *sstT* and GcvB are highlighted in yellow (*76, 77*).

A

GlnZ

MEME E-value: 3.9e-09

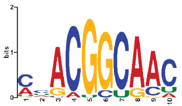

3'-...CUCUACCCGACUUUCAAA**GGUGCCGUUG**AUUUAGGGCCGCAACAACGC...-5'

B

```
                             1  glnA CDS end                                                                                   120
1 MG1655                     GTACTACAGCGTCtaa--------------------------------------------------------------------------------------------------------
2 Shigella_boydii            GTACTACAGCGTCTAA--------------------------------------------------------------------------------------------------------
3 EPEC                       GTACTACAGCGTCTAATAGTT---------------------------------------------------------------------------------------------------
4 EHEC                       GTACTACAGCGTCTAATAGTT---------------------------------------------------------------------------------------------------
5 Shigella_flexneri          GTACTACAGCGTCTAATAGTT---------------------------------------------------------------------------------------------------
6 Klebsiella_pneumoniae      GTACTACAGCGTCTAATTTTG-----CTGTAAATCCAGGGTA------------------------------------------------------------------------------
7 Citrobacter_farmeri        GTACTACAGCGTTTAATGATAATTAGTTGTAATTCCCGTAGATTTCTTATTGCAGCAAGGCGGCAATCGATGGAATCCCGATGAGCTTAGTCCACTAAGTGATTCGGGTGACTGAGAGCA
8 Salmonella_enterica        GTACTACAGCGTTTAA---TCGTATATTAAAAATCCGACGAATTTCGCGTTGCTGCAAGGCGGCAACTGAGCACATCCCCGGGAGCATAGATAGCGATGTGACCGGGGTAAGCGAAGGCA
  clustal                    ************ ***

                           121                                         binding  site                                           240
1 MG1655                     ---------------------------------GTGTTTTAGTTGCCGTGGAAACTTTTCGCCTGTCTCTGGCAGGCCTGGGATCGGTGGCAAGCACATCACGCCGGATGCGACGCAAAT
2 Shigella_boydii            ---------------------------------GTGTTTTAGTTGCCGTGGAAACTTTTCGCCTGTCTCTGGCAGGCCTGGGATCGGTGGCAAGCACATCACGCCGGATGCGACGCAAAT
3 EPEC                       ----GAAGTTGTACTACCCGGCGCAACAACGCCGGGATTTAGTTGCCGTGGAAACTTTCAGCCCATCTCTG-------------------------------------------------
4 EHEC                       ----GAAGTTGTACTACCCGGCGCAACAACGCCGGGATTTAGTTGCCGTGGAAACTTTCAGCCCATCTCTG-------------------------------------------------
5 Shigella_flexneri          ----GAAGTTGTACTACCCGGC-GCACAACGCCGGGATTTAGTTGCCGTGGAAACTTTCAGCCCATCTCTG-------------------------------------------------
6 Klebsiella_pneumoniae      ------TGTCGTGTTGTGGCGCGGGATGCCCGGGATATTTTGTTGCCGTGGAAACTTTCAGCCCATCTCAG-------------------------------------------------
7 Citrobacter_farmeri        GCCAACACCGCTGCGGTAAGAA--AGATACGGAGAATTTTAGTTGCCGTGGAAACTTTTAGCCCATCACAC-------------------------------------------------
8 Salmonella_enterica        GCCAACGCAGCAGCAGCGTGAAAGGCGTCAGGAGTTTTTGAGTTGCCGTGGAAACTTTCAGCCCATCCCAG-------------------------------------------------
  clustal                                                    *   **  ****************   ***  ** *

                           241                                                                                                   350
1 MG1655                     GCGTCTTATCCGGCCTACACGGTGATGATGTGGTAGGCCGGAGCAGGTGAGTCGCTCTCCAACGTGAAGTTTGTCAGCTATCTGTAGCCCATCTCTGCATGGGCTTTTTT
2 Shigella_boydii            GCGTCTTATCCGGCCTACACGGTGATGATGTGGTAGGCCGGAGCAGGTGAGTCGCTCTCCAACGTGAAGTTTGTCAGCTATCTGTAGCCCATCTCTGCATGGGCTTTTTT
3 EPEC                       -------------------------------------------------------------------------------------------------CATGGGCTTTTTT
4 EHEC                       -------------------------------------------------------------------------------------------------CATGGGCTTTTTT
5 Shigella_flexneri          -------------------------------------------------------------------------------------------------CATGGGCTTTTTT
6 Klebsiella_pneumoniae      -------------------------------------------------------------------------------------------------GATGGGCTTTTTT
7 Citrobacter_farmeri        -------------------------------------------------------------------------------------------------GATGGGCTTTTTT
8 Salmonella_enterica        -------------------------------------------------------------------------------------------------GATGGGCTTTTTT
  clustal                                                                                                                     ************
```

Fig. S4: Newly identified GlnZ in EPEC has conserved features
(A) MEME (*38*) was used to identify a common sequence motif in the target sequences of the newly identified sRNA GlnZ (derived from the 3'UTR of *glnA*). Targets under both activating and non-activating conditions were analyzed, the most statistically significant motif was taken, and its E-value is reported. The motif was further tested to see if it complements a sub-sequence of GlnZ by searching a match to the motif on the reverse complement sequence of the sRNA, using MAST (*78*) (pink region in GlnZ sequence). The motif is presented 5' to 3'. **(B)** Alignment of *glnA* 3'UTRs of selected Enterobacterial genera is shown below. Conserved nucleotides are marked by * with shading. The end of the *glnA* ORF is conserved (light blue) as well as the suggested binding site (pink). The stop codon of the K-12 MG1655 *glnA* sequences is in lower case letters for reference. The Rho-independent terminator is highlighted in grey. Arrows indicate the arms of the terminator stem. Note the first arm of the stem has a variable location.

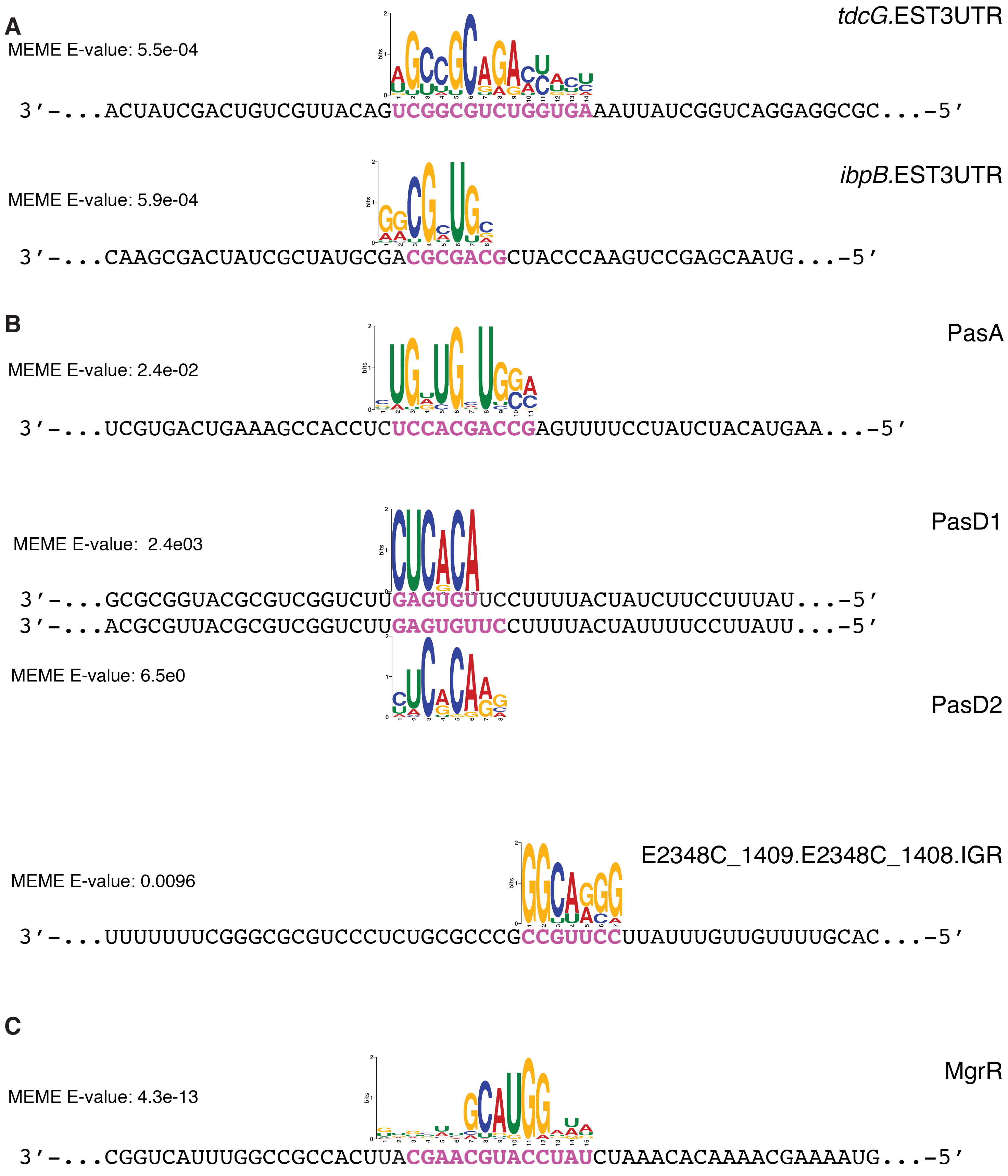
A
MEME E-value: 5.5e-04
tdcG.EST3UTR
3′-...ACUAUCGACUGUCGUUACAGUCGGCGUCUGGUGAAAUUAUCGGUCAGGAGGCGC...-5′
MEME E-value: 5.9e-04
ibpB.EST3UTR
3′-...CAAGCGACUAUCGCUAUGCGACGCGACGCUACCCAAGUCCGAGCAAUG...-5′
B
MEME E-value: 2.4e-02
PasA
3′-...UCGUGACUGAAAGCCACCUCUCCACGACCGAGUUUUCCUAUCUACAUGAA...-5′
MEME E-value: 2.4e03
PasD1
3′-...GCGCGGUACGCGUCGGUCUUGAGUGUUCCUUUUACUAUCUUCCUUUAU...-5′
3′-...ACGCGUUACGCGUCGGUCUUGAGUGUUCCUUUUACUAUUUUCCUUAUU...-5′
MEME E-value: 6.5e0
PasD2
MEME E-value: 0.0096
E2348C_1409.E2348C_1408.IGR
3′-...UUUUUUUCGGGCGCGUCCCUCUGCGCCCGCCGUUCCUUAUUUGUUGUUUUGCAC...-5′
C
MEME E-value: 4.3e-13
MgrR
3′-...CGGUCAUUUGGCCGCCACUUACGAACGUACCUAUCUAAACACAAAACGAAAAUG...-5′

Fig. S5. Common motifs in the target sets of newly identified sRNAs and MgrR are complementary to the putative sRNA sequence

As described in Supplementary Fig. S4, MEME (*38*) was used to identify a common sequence motif in the target sequences of newly identified sRNAs, and the motif was considered only if it was found to complement a sub-sequence of the sRNA. The latter was done by searching a match to the motif on the reverse complement sequence of the sRNA, using MAST (*78*), followed by manual evaluation. Putative binding sites on the sRNA are in bold pink. The motif is presented 5' to 3' and its E-value is reported. (**A**) Newly predicted sRNAs encoded in the core genome. (**B**) Newly identified sRNAs encoded in the accessory genome. (**C**) The common motif extracted for MgrR target sequences.

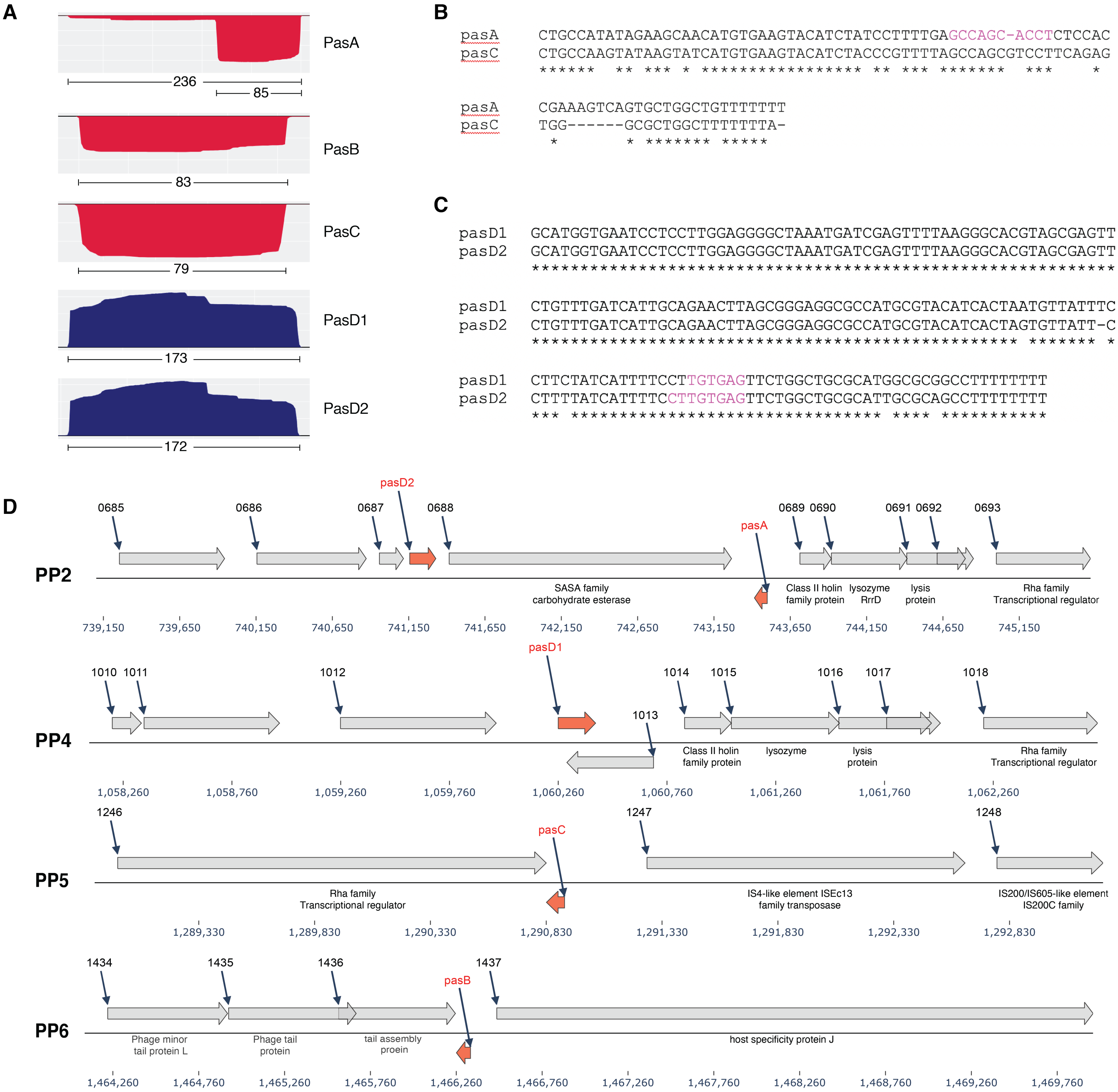

A
PasA
236
85
PasB
83
PasC
79
PasD1
173
PasD2
172
B
pasA CTGCCATATAGAAGCAACATGTGAAGTACATCTATCCTTTTGAGCCAGC-ACCTCTCCAC
pasC CTGCCAAGTATAAGTATCATGTGAAGTACATCTACCCGTTTTAGCCAGCGTCCTTCAGAG
pasA CGAAAGTCAGTGCTGGCTGTTTTTTT
pasC TGG------GCGCTGGCTTTTTTTA-
C
pasD1 GCATGGTGAATCCTCCTTGGAGGGGCTAAATGATCGAGTTTTAAGGGCACGTAGCGAGTT
pasD2 GCATGGTGAATCCTCCTTGGAGGGGCTAAATGATCGAGTTTTAAGGGCACGTAGCGAGTT
pasD1 CTGTTTGATCATTGCAGAACTTAGCGGGAGGCGCCATGCGTACATCACTAATGTTATTTC
pasD2 CTGTTTGATCATTGCAGAACTTAGCGGGAGGCGCCATGCGTACATCACTAGTGTTATT-C
pasD1 CTTCTATCATTTTCCTTGTGAGTTCTGGCTGCGCATGGCGCGGCCTTTTTTTT
pasD2 CTTTTATCATTTTCCTTGTGAGTTCTGGCTGCGCATTGCGCAGCCTTTTTTTT
D
PP2
0685
0686
0687
pasD2
0688
pasA
0689 0690
0691 0692
0693
SASA family carbohydrate esterase
Class II holin family protein
lysozyme RrrD
lysis protein
Rha family Transcriptional regulator
739,150 739,650 740,150 740,650 741,150 741,650 742,150 742,650 743,150 743,650 744,150 744,650 745,150
PP4
1010 1011
1012
pasD1
1013
1014
1015
1016
1017
1018
Class II holin family protein
lysozyme
lysis protein
Rha family Transcriptional regulator
1,058,260 1,058,760 1,059,260 1,059,760 1,060,260 1,060,760 1,061,260 1,061,760 1,062,260
PP5
1246
pasC
1247
1248
Rha family Transcriptional regulator
IS4-like element ISEc13 family transposase
IS200/IS605-like element IS200C family
1,289,330 1,289,830 1,290,330 1,290,830 1,291,330 1,291,830 1,292,330 1,292,830
PP6
1434
1435
1436
pasB
1437
Phage minor tail protein L
Phage tail protein
tail assembly proein
host specificity protein J
1,464,260 1,464,760 1,465,260 1,465,760 1,466,260 1,466,760 1,467,260 1,467,760 1,468,260 1,468,760 1,469,260 1,469,760

Fig. S6: Additional information about the newly identified Pathogenic Associated sRNAs.

(A) Coverage plots of PasA-D2 in one of the RNA-seq wild type libraries under the activating condition. For each Pas, the region overlapping the coordinates indicated in Supplementary Table S6, flanked by 10 nucleotides on each side, is drawn. Blue - sequences encoded on the 'plus' strand; red- sequences encoded on the 'minus' strand. Plots were drawn using an in-house developed genome browser. **(B-C)** Sequence alignment of PasA and PasC (**B**) and PasD1 and PasD2 **(C).** Predicted binding sites (Supplementary Fig. S5) are colored in magenta. **(D)** The genomic context of the *pas* genes. *pas* genes appear as red arrows while the other genes in the region are shown in grey with their locus tags written on top. Each line represents a different prophage in the EPEC accessory genome.

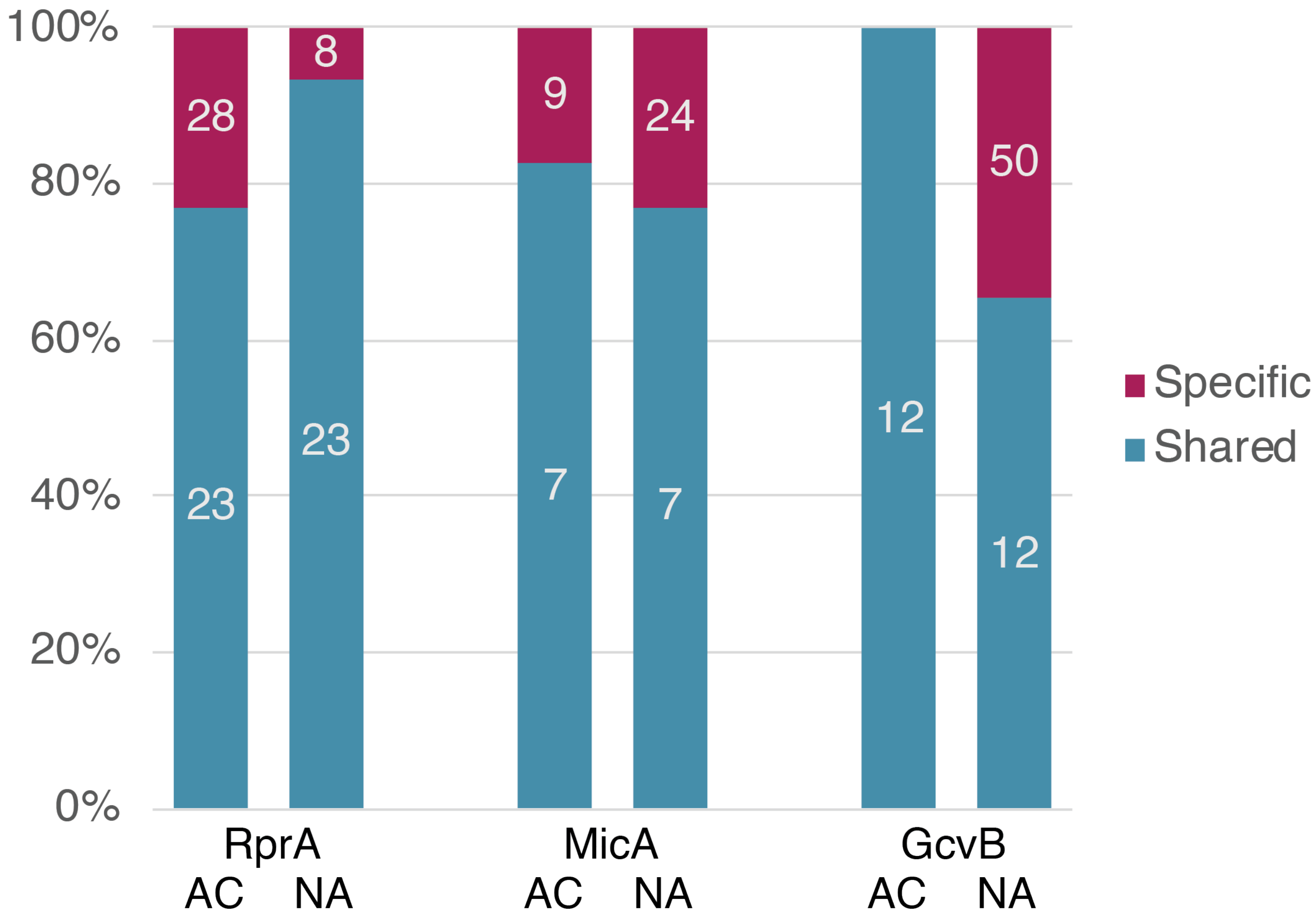

Fig. S7. Targets of sRNAs that exhibit similar abundances in both conditions. Presented are three sRNAs that exhibit similar relative fractions of S-chimeras in both activating and non-activating conditions (RprA: 0.06 and 0.04, MicA: 0.06 and 0.06, GcvB: 0.06 and 0.07; see Fig. 2 of the manuscript). For each sRNA the targets identified under each growth condition were divided into two groups: Shared – targets that were found to interact with the sRNA in both the activating and non-activating condition; Specific – targets that interacted with the sRNA only in the respective condition. Colored bar heights represent the relative fractions of S-chimeras associated with each sRNA, while the numbers indicate the number of different interactions (i.e., targets). For all three sRNAs most S-chimeras represent targets identified in the two conditions.

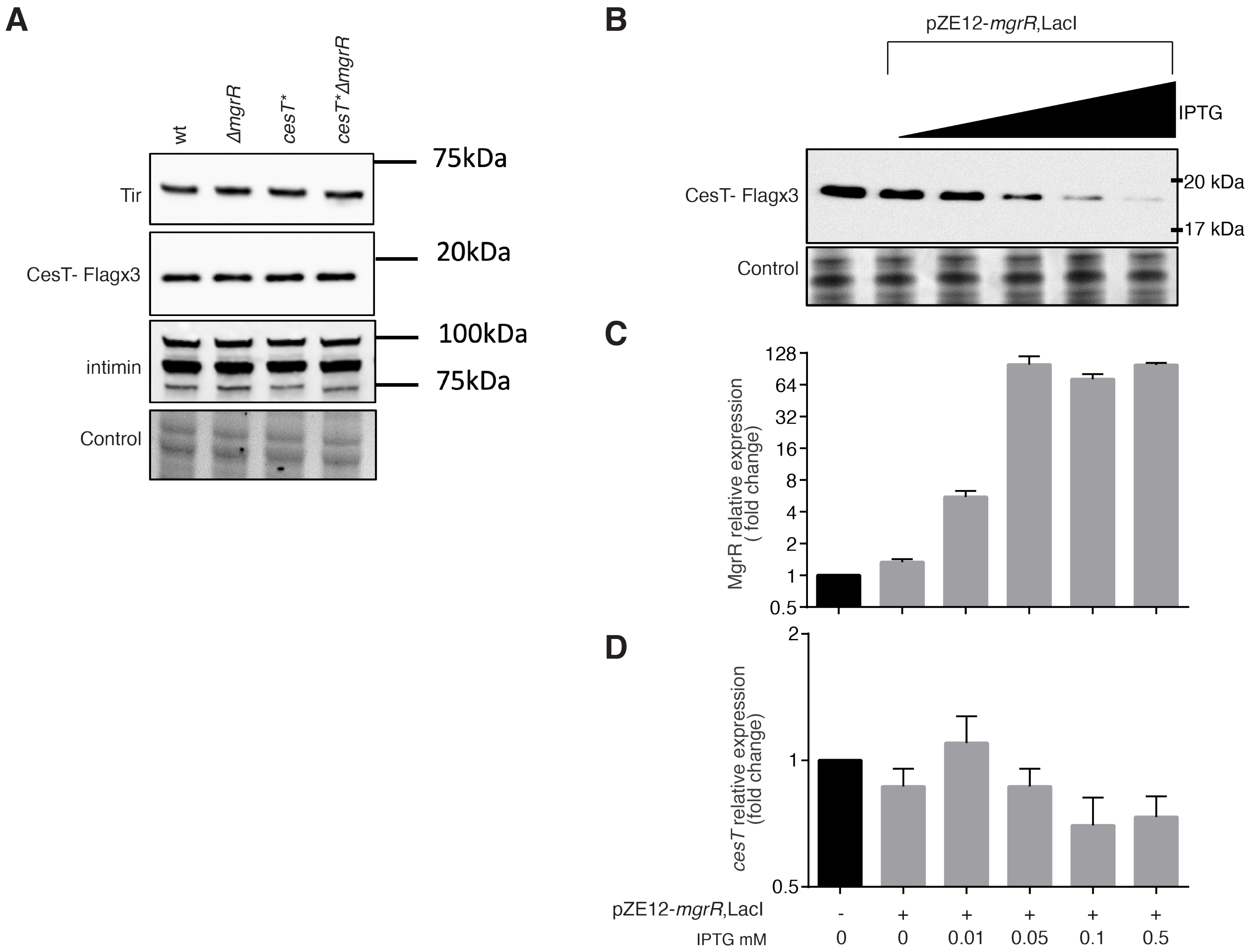

Fig. S8. MgrR inhibits CesT translation in a dose dependent manner

(A) Total protein was extracted from cultures of wild type EPEC, single mutants Δ*mgrR* and *cesT** and a double mutant *cesT**Δ*mgrR*, grown under the activating condition. Proteins were then analyzed by Western blot using antibodies raised against Tir, CesT and intimin. Stain-Free gel of total protein was used as a loading control. (B-D) Wild type EPEC containing a chromosomal Flag-tagged *cesT* was transformed with plasmids expressing MgrR and LacI (pZE12-MgrR, pREP4). Bacteria were statically grown overnight in LB, sub-cultured in DMEM, and grown for 2h to an OD_{600} of ~0.1. Next, cultures were treated with increasing concentrations of IPTG (final 0, 0.01, 0.05, 0.1 or 0.5 mM final) and grown for additional 2h to an OD_{600} of ~0.45. As a control, a culture of the same strain which did not carry the MgrR and LacI expressing plasmids was grown to the same growth phase without IPTG treatment. Culture samples were used for total protein and total RNA extraction. Note that the different lanes/bars are the same for B-D and their annotation appears below D. (B) CesT-Flag levels analyzed by western blotting. Coomassie staining of the total proteins was used as loading control. Increasing IPTG concentrations are indicated by black triangle. Protein size is indicated. (C) Quantitation of MgrR levels in total RNA using quantitative PCR. The value of MgrR level in the control sample (black column) was set as 1. Presented are the ratios between MgrR levels in each of the samples, normalized by 16S rRNA levels, and the control sample.

(D) Quantification of *cesT* mRNA levels in total RNA using quantitative PCR. The value of *cesT* mRNA level in the control sample (black column) was set as 1. Presented are the ratios between *cesT* mRNA levels in each of samples, normalized by 16S rRNA levels, and the control sample. Error bars indicate standard deviation of mean value of three biological repeats. The Y-axis in (C) and (D) is in $\log_2$ scale.

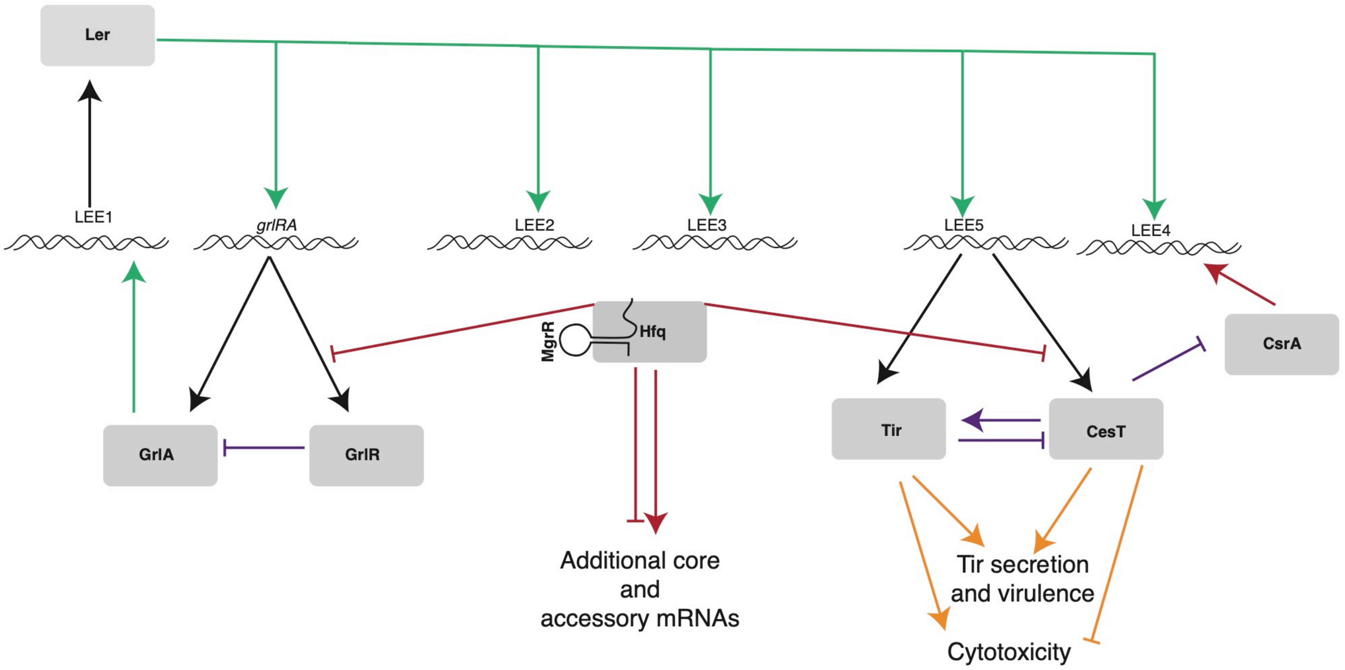

Supplementary Fig. S9: Graphical summary of EPEC virulence regulatory circuit related to MgrR

Wavy lines and grey boxes represent genes and proteins, respectively. Different colors of regulatory arrows represent different modes of action: Black-expression, green- transcription activation, red-posttranscriptional regulation, purple-protein-protein interaction and orange-action.
